# Cross-sectional associations between central and general adiposity with albuminuria: observations from 400,000 people in UK Biobank

**DOI:** 10.1038/s41366-020-0642-3

**Published:** 2020-07-16

**Authors:** Pengfei Zhu, Sarah Lewington, Richard Haynes, Jonathan Emberson, Martin J. Landray, David Cherney, Mark Woodward, Colin Baigent, William G. Herrington, Natalie Staplin

**Affiliations:** 1 Nuffield Department of Population Health (NDPH), Medical Research Council Population Health Research Unit at the University of Oxford, Oxford, UK; 2 Clinical Trial Service Unit and Epidemiological Studies Unit, NDPH, University of Oxford, Oxford, UK; 3 Oxford Kidney Unit, Churchill Hospital, Headington, Oxford, UK; 4 Division of Nephrology, Department of Medicine, Toronto General Hospital, University of Toronto, Toronto, ON Canada; 5 Department of Physiology and Institute of Medical Sciences, and Department of Pharmacology and Toxicology, University of Toronto, Toronto, ON Canada; 6 The George Institute for Global Health, University of Sydney, Sydney, NSW Australia; 7 The George Institute for Global Health, University of Oxford, Oxford, UK

**Keywords:** Risk factors, Epidemiology

## Abstract

**Background:**

Whether measures of central adiposity are more or less strongly associated with risk of albuminuria than body mass index (BMI), and by how much diabetes/levels of glycosylated haemoglobin (HbA1c) explain or modify these associations, is uncertain.

**Methods:**

Ordinal logistic regression was used to estimate associations between values of central adiposity (waist-to-hip ratio) and, separately, general adiposity (BMI) with categories of urinary albumin-to-creatinine ratio (uACR) in 408,527 UK Biobank participants. Separate central and general adiposity-based models were initially adjusted for potential confounders and measurement error, then sequentially, models were mutually adjusted (e.g. waist-to-hip ratio adjusted for BMI, and vice versa), and finally they were adjusted for potential mediators.

**Results:**

Levels of albuminuria were generally low: 20,425 (5%) had a uACR ≥3 mg/mmol. After adjustment for confounders and measurement error, each 0.06 higher waist-to-hip ratio was associated with a 55% (95%CI 53–57%) increase in the odds of being in a higher uACR category. After adjustment for baseline BMI, this association was reduced to 32% (30–34%). Each 5 kg/m^2^ higher BMI was associated with a 47% (46–49%) increase in the odds of being in a higher uACR category. Adjustment for baseline waist-to-hip ratio reduced this association to 35% (33–37%). Those with higher HbA1c were at progressively higher odds of albuminuria, but positive associations between both waist-to-hip ratio and BMI were apparent irrespective of HbA1c. Altogether, about 40% of central adiposity associations appeared to be mediated by diabetes, vascular disease and blood pressure.

**Conclusions:**

Conventional epidemiological approaches suggest that higher waist-to-hip ratio and BMI are independently positively associated with albuminuria. Adiposity–albuminuria associations appear strong among people with normal HbA1c, as well as people with pre-diabetes or diabetes.

## Background

The rising levels of adiposity in many regions in the world may partly explain the global ~30% increase in mortality directly attributed to chronic kidney disease (CKD) over the last decade [1]. Increased general adiposity, estimated from body mass index (BMI), is associated with higher risk of advanced CKD (i.e. stage 4 or 5) [2–5]. Compared with the apparent optimum BMI (20 to <25 kg/m^2^), advanced CKD risk is increased by about one-third in those who are overweight (BMI 25 to <30 kg/m^2^), is approximately doubled in early obesity (BMI 30 to <35 kg/m^2^); and is tripled at a BMI ≥ 35 kg/m^2^ [2]. Adiposity–CKD associations may result from concomitant dysglycaemia and raised blood pressure, but the relative contribution of general and central adiposity levels to determining level of these mediators may differ. For example, studies have found that BMI is a stronger predictor of blood pressure than central adiposity [6, 7], whilst measures of central adiposity are a stronger predictor of insulin resistance, diabetes status [8] and risk of acute myocardial infarction [9, 10] than BMI. However, the large prospective studies which reported strong positive associations between BMI and advanced CKD have not reported associations of central adiposity with advanced CKD for a given BMI [2, 3, 5, 11].

Albuminuria is a marker of early CKD, predicts progression and is used for CKD staging [12]. Its use as an outcome provides an opportunity to compare the independent associations between general and central adiposity levels with risk of early kidney disease. Prior to embarking on the presented adiposity–albuminuria analyses, we performed a systematic review and identified 46 published observational studies which have examined adiposity and albuminuria (search results and study details are provided in Supplemental Tables S1, S2). Of these 46 studies, only five developed models which considered central adiposity measures following adjustment for BMI. Two smaller studies (<5000 participants) found no association between central adiposity and proteinuria after adjusting for BMI [13, 14], two other small studies (both from Chinese populations) found a positive association [15, 16], whilst a large (>200,000) Japanese study found central adiposity to be associated with proteinuria after adjusting for BMI in men but not women [17]. Whether measures of central adiposity are more or less strongly associated with risk of albuminuria than BMI is therefore uncertain.

Whether adiposity–albuminuria associations are explained or modified by glycosylated haemoglobin (HbA1c) or dietary sodium is also uncertain. These are particularly relevant to consider following recent demonstrations of kidney protection using sodium-glucose co-transporter-2 (SGLT-2) inhibitors [18, 19], and glucagon-like peptide-1 receptor agonists [20, 21]. These therapies reduce body weight and HbA1c and also seem to impact intraglomerular haemodynamics, with consequent reductions in the risk of albuminuria and progressive CKD. They are also natriuretic which is relevant, as high dietary sodium has been suggested to confound adiposity–albuminuria associations [22].

UK Biobank is a prospective cohort of 500,000 UK adults designed for exploring the causes of complex diseases in middle-to-older age [23–25]. The baseline survey included systematic recording of several different measures of central and general adiposity and measurement of urinary albumin-to-creatinine ratio (uACR), as well as HbA1c and urinary sodium. Our objectives were to use the large size of UK Biobank to quantify precise associations between central and general adiposity measures with differences in the levels of albuminuria observed within an overweight general population, and to address current uncertainties.

## Materials and methods

### Study design and participants

During 2006–2010, 502,650 participants were recruited into UK Biobank at 22 UK-based assessment centres. Baseline assessments included a self-completed touch-screen questionnaire supplemented by interviews, standardised physical and functional measurements including bioimpedance, and the collection of biological samples. A subsample of participants has been subsequently re-surveyed. Full details of UK Biobank design have been described elsewhere [23–25].

In these analyses we excluded participants who withdrew their data (*n* = 133); and those with: self-reported cancer (*n* = 38,516), chronic obstructive pulmonary disease (*n* = 1,563), or liver cirrhosis/liver failure (*n* = 323), those with a BMI < 15 or >60 kg/m^2^ (*n* = 77); and those with no data on adiposity measures (*n* = 9,221), blood pressure (*n* = 1757), HbA1c (*n* = 30,878), urinary albumin (11,649) or urinary creatinine (*n* = 6). A total of 408,527 participants remained, including 190,386 men and 218,141 women (Supplemental Fig. S1).

### Exposures

The UK Biobank recorded four baseline measures of central adiposity: waist-to-hip ratio, waist-to-height ratio, waist circumference and percent trunk fat measured by bioimpedance [26]. Four measures of general adiposity were also recorded: BMI, height-adjusted weight, hip circumference and percent body fat. Standing height was measured using a Seca 202 telescopic height-measuring rod. Waist circumference at the level of the umbilicus and hip circumference were measured using a Wessex non-stretchable sprung tape measure. Weight, body fat (%) and trunk fat (%) were measured using a Tanita BC-418 MA body composition analyser. Further details are available in UK Biobank documentation [27]. Waist-to-hip ratio and BMI were selected as the primary focus of analyses due both to their widespread common use and low correlation between these two measures. All adiposity measures were categorised into fifths.

### Outcome

Some participants had no detectable albuminuria (i.e. the concentration was below the detectable limit of 6.7 mg/L). For those with detectable levels, uACR was calculated as a ratio of the paired albumin and creatinine measurements in the same urine sample. uACR was categorized according to the distribution of uACR or established cut-offs as follows: undetectable uACR, low “normal” (uACR 0.1 to <1 mg/mmol), high “normal” (uACR 1 to <3 mg/mmol) and albuminuria (uACR ≥ 3 mg/mmol) [12].

### Statistical analysis

In order to maximise the data and include a category for undetectable uACR, ordinal logistic regression was used to estimate the strength of the associations between each adiposity measure and uACR. To assess the shape of the associations for each adiposity measure, participants were divided into fifths (I–V) based on the given measure. For each category of the adiposity measures, an odds ratio (OR) relative to the bottom fifth was estimated using an ordinal logistic regression model. This gives the ratio of the odds of being in a higher uACR category (i.e. low normal vs undetectable, high normal vs low normal or albuminuria vs high normal). Sensitivity analyses using any albuminuria as a binary outcome (uACR ≥ 3 mg/mmol versus undetectable or “normal” levels) were also performed.

Potential confounders and effect mediators were identified based on the assumed pathways between the exposure (adiposity) and the outcome (uACR, see model assumptions in Supplemental Fig. S2). The separate relevance of each central and general adiposity measure to uACR was then assessed in confounder-adjusted models adjusted for baseline age (continuous), ethnicity (categorical: white, mixed, Asian or Asian British, Black or Black British, Chinese, other ethnic group, don’t know/missing), education (categorical: College/University degree, A levels/AS levels or equivalent, O levels/CSEs/NVQ/others, none of the above, prefer not to answer), Townsend deprivation index (categorical: fifths), smoking (categorical: current smoker, previous smoker, never smoker, prefer not to answer/missing), physical activity (categorical: <10MET-h/week, 10–49.9 MET-h/week, >50 MET-h/week, missing) and urinary sodium-to-creatinine ratio (Na^+^:Cr ratio, an estimate of recent dietary sodium intake: fifths) [28]. Adiposity-adjusted models were then constructed by mutually adjusting for a baseline central-general adiposity pair (e.g. waist-to-hip ratio was adjusted for BMI, and vice versa) to estimate the independent effect of each central and general adiposity measure. Finally, mediator-adjusted models were further adjusted for diabetes status, duration of diabetes and any prior history of vascular disease (myocardial infarction, angina, or stroke), and systolic/diastolic blood pressure (SBP/DBP) at baseline, to assess the extent to which any effect of adiposity on uACR is through these proposed mediators. Diabetes status was categorised as diabetes (self-reported diabetes [any type] or HbA1c ≥ 6.5%), pre-diabetes (HbA1c between 5.7 and <6.5%) and no diabetes (HbA1c < 5.7%).

Single baseline adiposity measurements are subject to random measurement error as well as possible changes over time. Failure to account for this leads to an underestimation of the true strength of the relationship (i.e. regression-dilution bias) [29]. Other work on adiposity suggests the vast majority of regression-dilution bias for the presented exposures relates to measurement error (as regression to the mean does not increase substantially over time), and that waist and hip circumference measurements are more susceptible to measurement error than BMI [30]. To ensure comparability between different adiposity measures, the mean of repeated adiposity measurements at resurvey (about 4.3 years after the baseline survey) in 16,833 participants was used as the estimate of measurement-error adjusted adiposity levels for individuals in each baseline adiposity fifth. The results of a sensitivity analysis without any correction for potential measurement error are provided for comparison.

Since there was a generally log-linear association between adiposity and log odds of higher uACR level for the top four baseline adiposity fifths, the OR per incremental increase in adiposity value was calculated as the exponential of the slope of the inverse variance weighted regression through these four log ORs. ORs per 5 kg/m^2^ are provided for both sexes, which equates to 1.10× the standard deviation (SD) for BMI in the study population. ORs for other adiposity measures were scaled accordingly as follows: waist-to-hip ratio per 0.06; waist-to-height ratio per 0.07; waist circumference per 12.1 cm; percent trunk fat per 7.3%; height-adjusted weight per 14 kg; hip circumference per 9.2 cm; percent body fat per 7.6% (Note: SDs are reduced after taking account of measurement error, which was corrected by multiplying the SD by the square root of the regression-dilution ratio). All analyses were performed for men and women separately with overall estimates calculated by taking an inverse variance weighted average of the sex-specific estimates. Subgroup analyses by particular characteristics were performed on the model mutually adjusted for a central/general adiposity measure at baseline (i.e. adiposity-adjusted models).

The proportional odds assumption was checked for all covariates used in the analyses, including each of the adiposity measures. For urinary sodium-to-creatinine ratio, the assumption was found to be violated. To assess whether urinary sodium-to-creatinine ratio was an important confounder of associations, the ORs for adiposity in a confounder-adjusted proportional odds model (not including urinary sodium-to-creatinine ratio) were compared with those from a partial proportional odds model (with the proportional odds relaxed for urinary sodium-to-creatinine), and a likelihood ratio test used to assess for any significant improvement in model fit. No important confounding effect was noted so urinary sodium-to-creatinine was omitted from models based on ordinal logistic regression. All analyses used SAS version 9.4 (SAS Institute, Cary NY, USA) and R version 3.5.1.

## Results

### Baseline characteristics

The mean age of the 408,527 included participants was 56.2 years (SD 8.1) (Table 1). Ninety-four percent (*n* = 385,897) of participants were white, and nearly one-third (*n* = 133,155) had obtained a college or university degree. Nine percent of the participants had diabetes (*n* = 23,637) or pre-diabetes (*n* = 12,507) and 5.5% (*n* = 22,318) had a history of vascular disease. Mean SBP was 138 (SD 19) mmHg.

**Table 1:** Baseline characteristics of UK Biobank, overall and by sex.

Characteristics	Men (*n* = 190,386)	Women (*n* = 218,141)	All (*n* = 408,527)
Exposure			
Adiposity			
Waist-to-hip ratio	0.93 (0.06)	0.82 (0.07)	0.87 (0.09)
Waist-to-height ratio	0.55 (0.06)	0.52 (0.08)	0.53 (0.07)
Waist circumference (cm)	97 (11)	84 (12)	90 (13)
Trunk fat (%)	27.6 (6.6)	34.0 (7.8)	31.0 (7.9)
BMI (kg/m²)	27.8 (4.2)	27.0 (5.1)	27.4 (4.7)
Height-adjusted weight (kg)	85.9 (12.9)	71.2 (13.3)	78.0 (15.0)
Hip circumference (cm)	103 (8)	103 (10)	103 (9)
Body fat (%)	25.2 (5.8)	36.5 (6.9)	31.2 (8.5)
Confounders			
Socio-demographics			
Age, years	56.4 (8.2)	56.1 (8.0)	56.2 (8.1)
White	179,611 (94.3%)	206,286 (94.6%)	385,897 (94.5%)
College or University degree	64,727 (34.0%)	68,428 (31.4%)	133,155 (32.6%)
Townsend deprivation score	−2.2 (−3.7, 0.5)	−2.2 (−3.7, 0.4)	−2.2 (−3.7, 0.5)
Lifestyle			
Current smoker	23,554 (12.4%)	19,084 (8.7%)	42,638 (10.4%)
Daily drinker	48,044 (25.2%)	35,225 (16.1%)	83,269 (20.4%)
Physical activity, MET-h/week	34.5 (36.3)	30.1 (30.2)	32.2 (33.3)
Urinary sodium-to-creatinine ratio (mmol/mmol)^a^	8.8 (6.0, 12.3)	10.4 (6.9, 14.9)	9.6 (6.4, 13.7)
Mediators			
Health status			
Diabetes^b^	14,693 (7.7%)	8,944 (4.1%)	23,637 (5.8%)
HbA1c (%)	6.8 (6.2, 7.6)	6.7 (6.2, 7.5)	6.8 (6.2, 7.6)
Duration of diabetes, years	4.0 (1.0, 10.0)	3.0 (0.0, 8.0)	4.0 (0.0, 9.0)
Pre-diabetes^b^	6,058 (3.2%)	6,449 (3.0%)	12,507 (3.1%)
HbA1c (%)	6.1 (6.1, 6.3)	6.1 (6.0, 6.2)	6.1 (6.0, 6.2)
No diabetes^b^	169,635 (89.1%)	202,748 (92.9%)	372,383 (91.2%)
HbA1c (%)	5.3 (5.1, 5.5)	5.3 (5.1, 5.5)	5.3 (5.1, 5.5)
Any vascular disease^c^	15,366 (8.1%)	6,952 (3.2%)	22,318 (5.5%)
Systolic blood pressure (mmHg)	141 (17)	135 (19)	138 (19)
Diastolic blood pressure (mmHg)	84 (10)	81 (10)	82 (10)
eGFR (mL/min/1.73 m²)^d^	90.9 (13.1)	91.4 (13.2)	91.2 (13.2)
Outcome			
Urinary albumin-to-creatinine ratio (mg/mmol)^a^	0.9 (0.6, 1.9)	1.2 (0.7, 2.2)	1.0 (0.6, 2.0)
Undetectable albumin	123,986 (65.1%)	157,894 (72.4%)	281,880 (69.0%)
≥0.1 to <1	35,867 (18.8%)	25,207 (11.6%)	61,074 (14.9%)
≥1 to <3	20,300 (10.7%)	24,848 (11.4%)	45,148 (11.1%)
≥3	10,233 (5.4%)	10,192 (4.7%)	20,425 (5.0%)

Arithmetic mean (SD), *N* (%) or median (Q1, Q3) shown, unless otherwise stated.

Exclusion criteria: participants with self-reported cancer, chronic obstructive pulmonary disease or liver failure/cirrhosis; or participants with missing values of adiposity measures, blood pressure, HbA1c, or urinary albumin-to-creatinine ratio.

*BMI* body mass index, *HbA1c* glycosylated haemoglobin, *eGFR* estimated glomerular filtration rate

^a^Median (Q1, Q3) among those with detectable values.

^b^Diabetes is defined as self-reported diabetes or HbA1c ≥ 6.5%, pre-diabetes is defined as HbA1c between 5.7 and <6.5%, no diabetes is defined as HbA1c < 5.7%.

^c^Any vascular diseases include heart attack, angina, or stroke.

^d^eGFR was calculated from the Chronic Kidney Disease Epidemiology Collaboration (CKD-EPI) creatinine equation.

For both men and women, the lowest correlation was between waist-to-hip ratio and BMI (men, *ρ* = 0.60; women, *ρ* = 0.46). Age-adjusted correlations between BMI and other three adiposity markers (waist circumference, waist-to-height ratio and trunk fat %) were stronger (all *ρ* > 0.75) and similar in men and women (Supplemental Fig. S3).

Mean waist-to-hip ratio and BMI were 0.87 (0.09) and 27.4 (4.7) kg/m^2^, respectively, and were slightly higher in men than women (sex differences were more marked for waist-to-hip ratio [0.93 versus 0.82], Table 1/Supplemental Tables S3, 4). Higher levels of age-adjusted waist-to-hip ratio and BMI were also observed in men and women with lower educational qualifications, from more deprived areas, with lower levels of physical activity, who ever smoked, and if they reported diabetes or vascular disease (Supplemental Table S5).

Two-thirds of men (*n* = 123,986) and nearly three-quarter of women (*n* = 157,894) had no detectable urinary albumin. Among those with detectable uACR, median uACR was 1.0 (IQR 0.6, 2.0) mg/mmol. The crude prevalence of albuminuria (uACR ≥ 3 mg/mmol) was 5.4% (*n* = 10,233) in men and 4.7% (*n* = 10,192) in women (Table 1).

### Waist-to-hip ratio

**Fig. 1:**
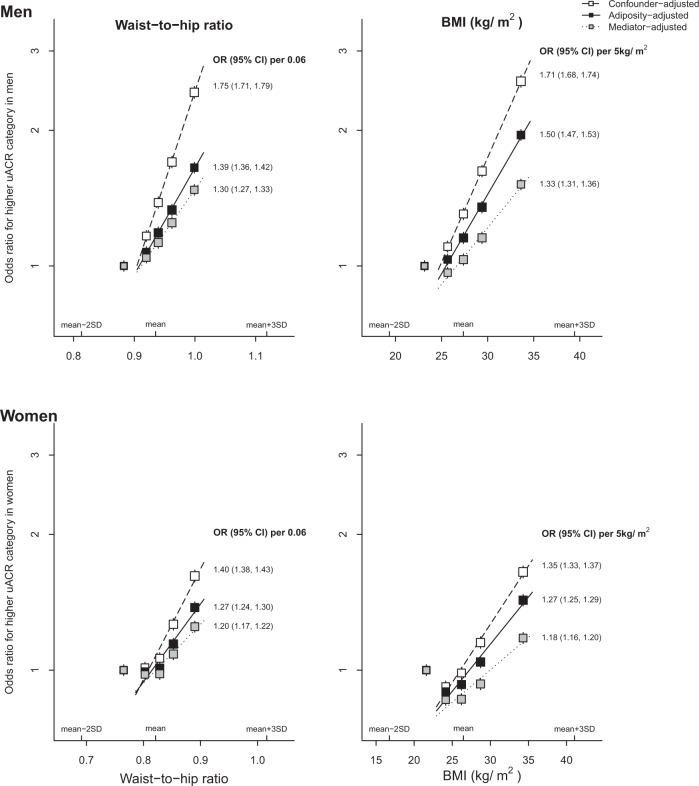
Associations between adiposity (waist-to-hip ratio and BMI) and a higher urinary albumin-to-creatinine ratio category by sex. BMI body mass index, uACR urinary albumin-to-creatinine ratio, SBP systolic blood pressure, DBP diastolic blood pressure. Confounder-adjusted model: adjusted for age, ethnicity, education, region, Townsend deprivation index, smoking, physical activity at baseline. Adiposity-adjusted model: further adjusted for reciprocal adiposity (i.e. waist-to-hip ratio adjusted for BMI, and BMI adjusted for waist-to-hip ratio) at baseline. Mediator-adjusted model: further adjusted for diabetes status (diabetes, pre-diabetes, no diabetes), duration of diabetes, SBP, DBP and any self-reported vascular disease (heart attack, angina and stroke) at baseline.

Within-person variability for waist-to-hip ratio was higher than for BMI (Supplemental Table S6). After adjustment for confounders and measurement error, the association between waist-to-hip ratio and uACR was “J”-shaped, but log-linear if the lowest category of waist-to-hip ratio was excluded (Fig. 1). For men and women respectively, each 0.06 higher waist-to-hip ratio was associated with a 75% (71–79%) and 40% (38–43%) increase in odds of higher uACR. For both sexes combined, each 0.06 higher waist-to-hip ratio was associated with a 55% (53–57%) increase in odds. This association attenuated after adjustment for baseline BMI to a 32% (30–34%) increase in odds. The odds of higher uACR level reduced to 24% (22–26%) after further adjustment for diabetes status, duration of diabetes, vascular disease and blood pressure, with model chi square values reduced from 1,297 to 770 (Supplemental Fig. S4). These results were similar when albuminuria was used as a binary outcome (Supplemental Fig. S5).

### Body mass index

Like waist-to-hip ratio, the confounder-adjusted model found associations between BMI and uACR which were “J”-shaped, but were log-linear if the lowest category was excluded (Fig. 1). For men and women, a 5 kg/m^2^ higher BMI was associated with 71% (68–74%) and 35% (33–37%) increased odds of higher uACR, respectively (Fig. 1). For both sexes combined, each 5 kg/m^2^ higher BMI was associated with a 47% (46–49%) increased odds of a higher uACR level. This association attenuated to a 35% (33–37%) increased odds after adjustment for baseline waist-to-hip ratio. After further adjustment for mediators, each 5 kg/m^2^ higher BMI adjusted for waist-to-hip ratio was associated with a 23% (22–25%) increase in odds of higher uACR, with model chi square values reduced from 2,731 to 1,278 (Supplemental Fig. S4).

Analogous analyses for other adiposity exposures in adiposity-adjusted models (i.e. models assessing a central adiposity measure adjusted for a general adiposity measure, or vice versa) showed a broadly similar pattern, with the exception of waist circumference associations, which increased after adjustment for hip circumference, and hip circumference associations which were not associated with albuminuria once models had adjusted for waist circumference (Supplemental Tables S7a, b and Supplemental Fig. S6). Supplemental Fig. S7 provides sensitivity analyses without adjustment for measurement error.

### Effects by diabetes status

**Fig. 2:**
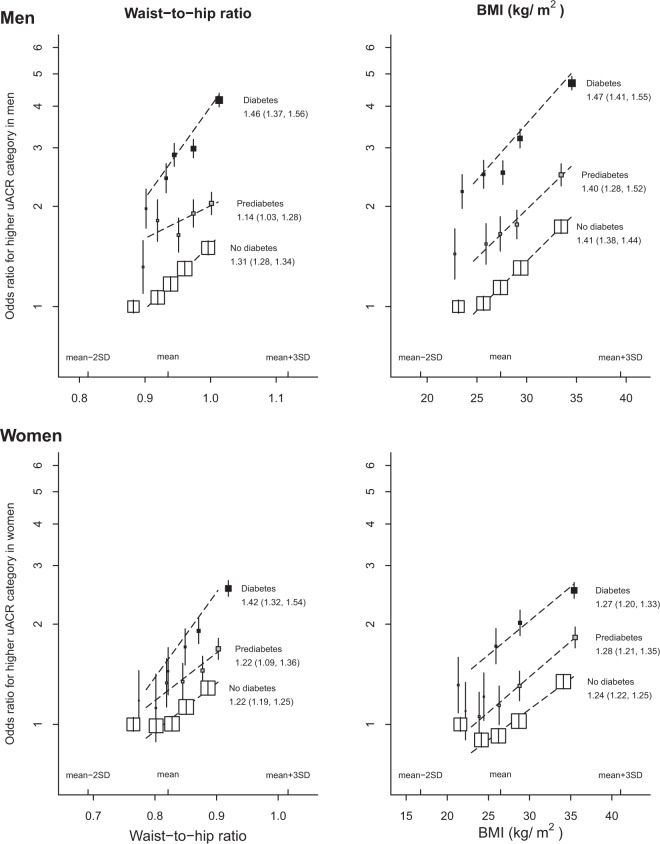
Associations between adiposity (waist-to-hip adjusted for BMI and BMI adjusted for waist-to-hip ratio) and a higher urinary albumin-to-creatinine ratio category by sex and by diabetes status. BMI body mass index, uACR urinary albumin-to-creatinine ratio. Models were adjusted for confounders (age, ethnicity, education, region, Townsend deprivation index, smoking, physical activity) and a reciprocal adiposity measurement (i.e. waist-to-hip ratio adjusted for BMI, BMI adjusted for waist-to-hip ratio).

**Fig. 3:**
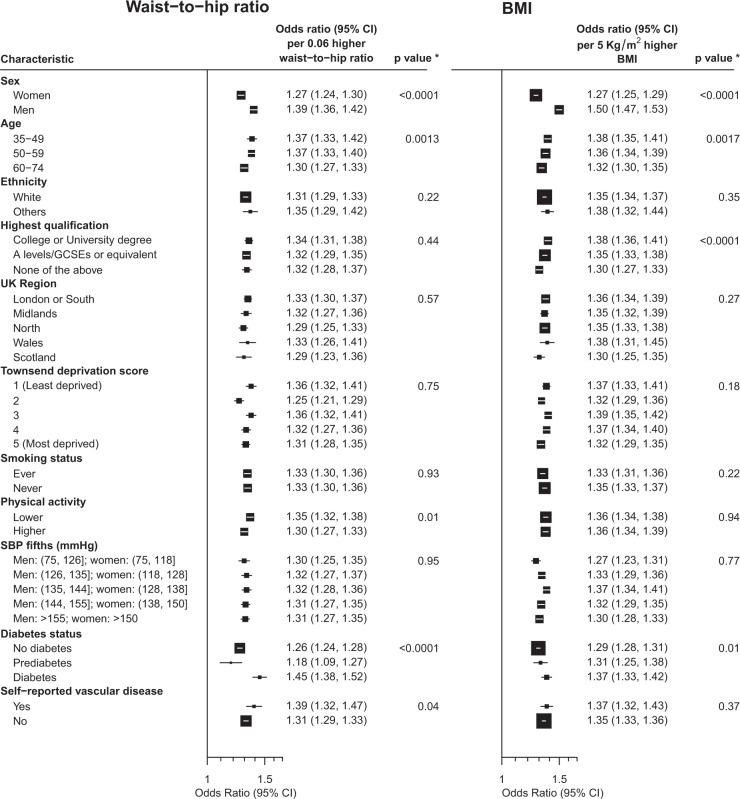
Associations between incremental increase in adiposity (waist-to-hip ratio adjusted for BMI and BMI adjusted for waist-to-hip ratio) and a higher urinary albumin-to-creatinine ratio category by participant characteristics. *p value for trend or heterogeneity test. BMI body mass index, SBP systolic blood pressure. Log ORs for a higher uACR category per incremental increase in adiposity by subgroups are the inverse variance weighted averages of the sex-specific log ORs by subgroups (calculated as the slopes of the inverse variance weighted regressions through the log ORs of the top four adiposity categories in each subgroup). Model was adjusted for confounders (age, ethnicity, education, region, Townsend deprivation index, smoking, physical activity where relevant) and reciprocal adiposity (i.e. waist-to-hip ratio adjusted for BMI, BMI adjusted for waist-to-hip ratio) at baseline.

Since the presence of albuminuria in a person with diabetes may reflect a different disease process to a person without diabetes, we assessed in more detail the shape of sex-specific associations by diabetes status. Figure 2 demonstrates that diabetes status increased the odds of being in a higher uACR category by 2–3-fold, with pre-diabetes status being intermediate risk between diabetes and no diabetes. There was some statistical evidence that both waist-to-hip ratio and BMI appeared to be somewhat more strongly associated with uACR in those with diabetes compared with those with pre-diabetes or without diabetes (*P*_trend_ < 0.0001 and 0.01, respectively, Fig. 3), but positive log-linear associations between both waist-to-hip ratio and BMI (after mutual adjustment) with higher uACR remained irrespective of diabetes status (Fig. 2).

### Effects by urinary sodium-to-creatinine ratio

Including urinary sodium-to-creatinine ratio in models improved fit (*p* < 0.001), but any confounding effect was small, as adiposity–albuminuria associations were changed little by its inclusion. The ORs per 5 kg/m^2^ higher BMI were 1.47 (1.43–1.50) versus 1.46 (1.43–1.50) before and after adjustment for urinary sodium-to-creatinine ratio, and per 0.06 higher waist-to-hip ratio were 1.62 (1.58–1.67) versus 1.62 (1.57–1.67), respectively. In women but not men, there was a suggestion that higher urinary sodium-to-creatinine ratio increased the odds of being in a higher uACR category (Supplemental Fig. S8). Nevertheless, in both men and women, the shape and size of associations between waist-to-hip ratio and BMI (after mutual adjustment) with higher categories of uACR appeared to be similar in those with the highest and lowest halves of urinary sodium-to-creatinine ratio.

### Effects by other characteristics

Other than sex and diabetes status, the only other consistent effect modifier was age. Those who were younger appeared to have somewhat stronger associations between waist-to-hip ratio and BMI (after mutual adjustment) with increased odds of higher levels of uACR than those who were older (*P*_trend_ = 0.0013 and 0.0017, respectively; Fig. 3).

## Discussion

UK Biobank provides an opportunity to estimate precise associations between a range of different measures of adiposity and albuminuria in middle-aged population which was, on average, overweight. There were four main findings from these analyses. First, the study provides the clearest evidence about the independent relevance of central adiposity to risk of albuminuria to date: increasing levels of central adiposity is associated with higher levels of albuminuria independent of measures of general adiposity. Each standard deviation higher waist-to-hip ratio appears to be as important as each standard deviation higher BMI. Secondly, although these associations are stronger in men than women, they clearly exist in both sexes. Thirdly, dietary sodium (estimated using urinary sodium-to-creatinine ratio) does not appear to confound adiposity–albuminuria associations. Lastly, adiposity associations were clearly evident in those without diabetes as well as those with pre-diabetes and diabetes, and models which included diabetes, blood pressure and vascular disease showed that such factors altogether explained about 40% of the observed central adiposity–albuminuria associations.

The associations between both waist-to-hip ratio and BMI with higher levels of albuminuria may represent early disturbances in intraglomerular haemodynamics resulting from excess adiposity. General population data with iohexol measured glomerular filtration rate have demonstrated that central adiposity [31] and impaired fasting glucose [32] among people without diabetes are associated with measured hyperfiltration (which is considered to be a precursor to albuminuria and CKD development). Such data suggest adiposity and small changes in glycaemic control can alter intraglomerular haemodynamics and may therefore conceivably affect albuminuria measurements within the “normal range”. Disturbances of tubuloglomerular feedback though upregulating tubular SGLT-2 function is one potential mechanism [33]. However, if this was the key mechanism, one would expect adiposity–albuminuria associations to be substantially weaker among those with normal HbA1c, and this does not appear to be the case (Fig. 2). Our findings extend and are consistent with other studies which have found the relative strength of the association between BMI and risk of advanced CKD risk is similar in shape and size to people with and without diabetes [2, 3, 5]. Taken together with randomised trial evidence [34], these conventional observational analyses suggest important pathways independent of glycaemic control and systemic blood pressure could be responsible for mediating some of adiposity-related renal risk. Such pathways may include altered intrarenal haemodynamics, perhaps through activation of the renin–angiotensin–aldosterone system, and potentially augmented levels of pro-inflammatory cascades [33].

In addition to metabolism-related pathways, renal sodium handling has been implicated in the pathogenesis of glomerular hyperfiltration and CKD progression [35]. A previous study using 24-h urinary collections to estimate dietary sodium and identify hyperfiltration raised a hypothesis that high dietary sodium may confound associations between adiposity and hyperfiltration [22]. The presented analyses did not confirm this hypothesis: no pathophysiological synergism between the effects of obesity [36] and high dietary sodium on albuminuria was evident with adiposity–albuminuria associations unmodified by levels of urinary sodium (Supplemental Fig. S8). These findings are consistent with results of trials of salt-restriction which have not demonstrated any clear effect of low dietary salt on proteinuria or changes in renal function [37].

The presented analyses support the need to tackle the high prevalence of overweight and obesity which is high and rising in many areas of the world [38, 39]. In the United Kingdom, prevalence of obesity (BMI ≥ 30 kg/m^2^) increased from 15 to 27% between 1993 and 2015 [40]. For those that are obese and have already developed albuminuria, low calorie diets and increased physical activity (or bariatric surgery) address the root cause and may reverse glomerular hyperfiltration and hypertension [34]. Lowering intraglomerular pressure using renin–angiotensin system inhibitors may be a useful therapeutic strategy, and SGLT-2 inhibition may have a broader range of renoprotective effects including reductions in body weight, hyperglycaemia as well as intraglomerular hypertension. Their effects on CKD progression outcomes are being assessed in large-scale CKD trials, including people without diabetes [41].

The present UK Biobank study is at least 10-times larger than any of the other reported studies of a Caucasian population to consider central adiposity and/or general measures with levels of albuminuria (Supplemental Table S2). However, there are some limitations. First, the cross-sectional and observational nature of the data limits assessment of the temporality of associations precluding assessment of causality. Secondly, measurement error in covariates in the model may lead to an underestimate of the importance of meditators (particularly blood pressure). Future analyses using genetically determined adiposity and Mendelian randomisation approaches could be used to address both these issues. Thirdly, although albuminuria is a strong predictor of advanced CKD [42] and is used to define and stage CKD in people with and without diabetes [12], it is not as important to patients as the need for renal replacement therapy. These novel findings therefore still need to be tested in prospective cohorts which are large and long enough to have recorded a sufficient number of incident end-stage kidney disease outcomes [5]. Fourthly, the use of spot samples to estimate dietary sodium intake has recognised limitations, but longer duration urine collections are less feasible in large-scale studies [43]. Lastly, UK Biobank is predominately a study of Caucasians and may not extrapolate to other populations around the world.

In conclusion, both higher central and general adiposity are independently associated with albuminuria and appear to be similarly important. Dietary sodium levels (estimated by urinary sodium) do not appear to confound these associations, and although increasing HbA1c clearly increases risk of albuminuria, adiposity–albuminuria associations appear strong among people with normal or elevated levels of HbA1c. As diabetes, blood pressure and prior vascular disease appear to explain about 40% of the central adiposity–albuminuria association, genetic epidemiological approaches and experimental work should be used to assess whether other mechanisms might underpin these observed associations.

## Supplementary information

Supplemental Materials
