# Supplementary material for: Cross-sectional associations between central and general adiposity with albuminuria: observations from 400,000 people in UK Biobank
Source: Int J Obes (Lond). 2020 Jul 16;44(11):2256–66. doi: 10.1038/s41366-020-0642-3 (PMC7577847; doi:10.1038/s41366-020-0642-3)
Supplement: Supplementary file 1 — Supplemental Materials [file 41366_2020_642_MOESM1_ESM.pdf]

Table S1: Literature review search terms and study selection flow sheet

Search criteria	Search terms (MeSH terms, or key word searches)
Adiposity	Adiposity OR obesity OR overweight OR anthropometry OR body distribution OR body composition OR body mass index OR body mass OR BMI OR height adjusted weight OR height-adjusted weight OR HtaW OR hip circumference OR waist circumference OR abdominal girth OR bio-impedance OR bioimpedance OR bioelectrical impedance OR body fat OR body fat percentage OR waist hip ratio OR waist:hip ratio OR waist-hip ratio OR waist-to-hip ratio OR waist height ratio OR waist:height ratio OR waist-height ratio OR waist-to-height ratio OR impedance OR trunk fat OR trunk fat percentage OR trunk body fat OR body fat distribution OR fat distribution OR subcutaneous fat distribution
Chronic kidney disease	Glomerular filtration rate OR GFR OR eGFR OR albuminuria OR microalbuminuria OR albumin OR urine albumin OR albumin to creatinine ratio OR albumin-to-creatinine ratio OR ACR OR uACR OR proteinuria OR protein to creatinine ratio OR protein creatinine ratio OR uPCR OR protein-to-creatinine ratio OR PCR OR albumin excretion rate OR AER OR uAER OR kidney disease OR chronic kidney disease OR CKD OR chronic renal disease OR renal progression OR renal function OR kidney function OR end stage renal disease OR end-stage renal disease OR ESRD OR kidney failure OR renal failure
Study design	Cross-sectional OR cross sectional OR observational OR case-control OR case control OR prospective OR longitudinal OR cohort OR genetic OR Mendelian randomisation OR Mendelian randomization

Databases and date range: Medline (from 1946 to 22/05/2018); Embase (from 1974 to 22/05/2018)

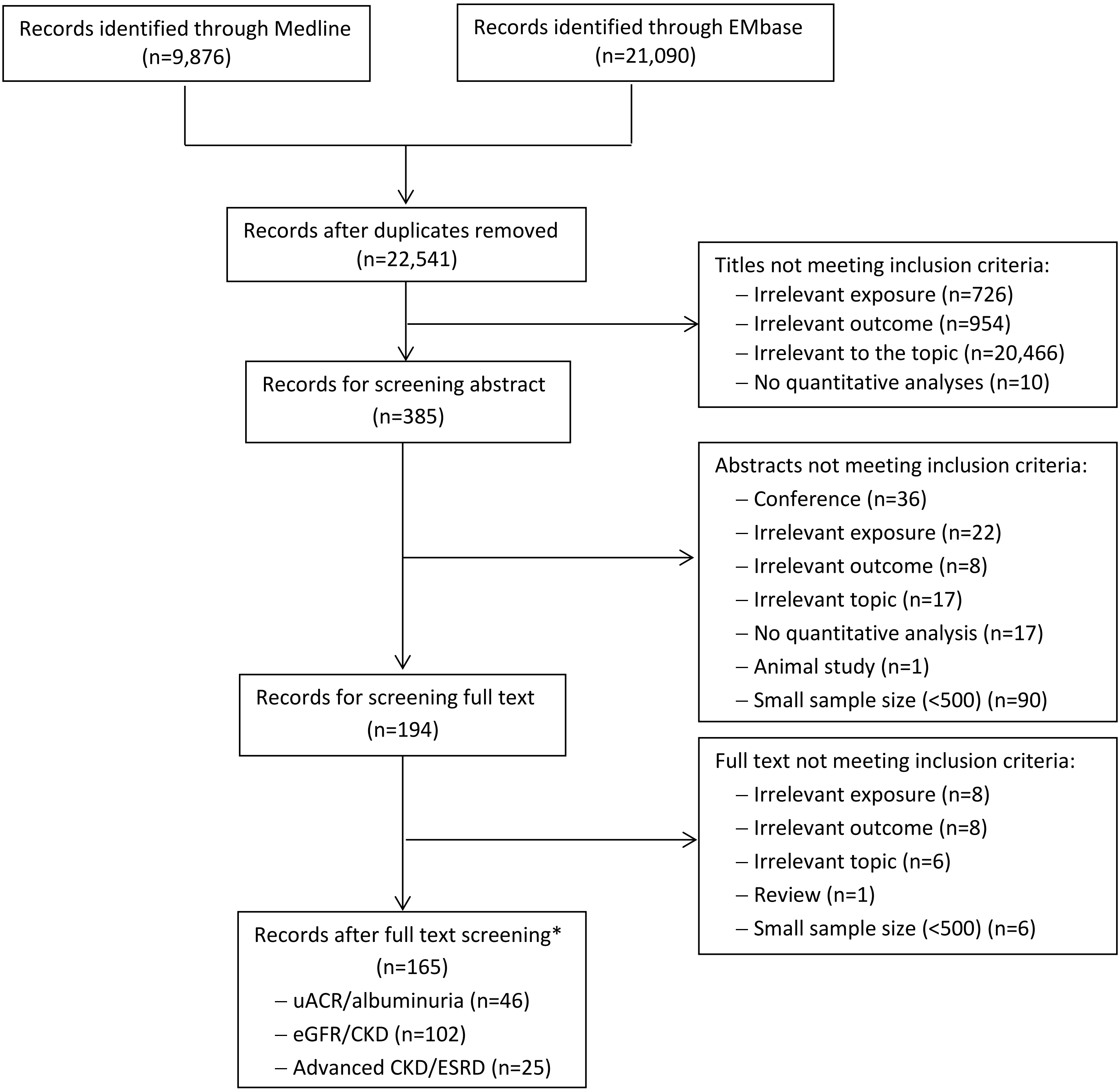

uACR: urinary albumin-to-creatinine ratio; eGFR: estimated glomerular filtration rate; CKD: chronic kidney disease; ESRD: end-stage renal disease.

* All studies included have been reviewed but this study summarises literatures on uACR or albuminuria outcomes.

Table S2: Literature review results (limited to studies using albuminuria as an outcome)

Study Baseline Characteristics								Study Results		
Study	Recruitment year (follow-up)	Study location	Sample size	Demographics	Co-morbidity prevalence	Adiposity measures	Outcome	Adjustments	Exposure	Reported association (ORs/HRs with 95% CI or β [p-value] shown)*
General adiposity										
Cohort studies										
Jang et. al 2014(1)	2002-2009	Korea	53,876	Age: 35<br>Male: 63%<br>Ethnicity: Korean	DM: 1.5%<br>HTN: 11.3%<br>SBP: N/A	BMI: 23.2 Kg/m^2<br>WC: 78.9 cm	Proteinuria	Demographics, lifestyle, DM, HTN, lipids, eGFR	BMI: cut-offs (18.5, 23, 25 Kg/m^2)	Obese vs normal weight:<br>Men: 1.38 (1.09-1.75)<br>Women: non-significant
Cederholm et al. 2004(2)	1997<br>(4.6 years)	Sweden	10,610	Age: 53<br>Male: 53%<br>Ethnicity: N/A	DM: 100%<br>HTN: N/A<br>SBP: 139 mmHg	BMI: 26.8 Kg/m^2	Microalbuminuria: urine albumin 20-200 µg/min	Demographics, lifestyle, BP, lipids, HbA1c	BMI:<br>per 1-Kg/m^2	T1DM: 5.93 (2.22-15.7)<br>T2DM: 2.03 (1.04-3.97)
Lin et al. 2012(3)	2004.10<br>(2009.7)	Taiwan	2,350	Age: 57<br>Male: 49%<br>Ethnicity: Chinese	DM: 12.1%<br>HTN: N/A<br>SBP: 136 mmHg	BMI: 24.3 Kg/m^2<br>WC: 81.4 cm	Albuminuria	Demographics, lifestyle, BP, lipids, eGFR, BMI	BMI	Non-significant
Rossi et al. 2010(4)	N/A	Italy	1,019	Age: 63<br>Male: 64%<br>Ethnicity: N/A	DM: 100%<br>HTN: 62%<br>SBP: N/A	BMI: 29.2 Kg/m^2<br>WC: 101.3 cm	Progression of UAE: doubling in ACR from baseline to follow up	Multivariable analysis with stepwise selection	BMI	Overall: 2.19 (1.16-4.15)
Tozawa et al. 2002(5)	1997<br>(1999)	Japan	5,403	Age: 48<br>Men: 63%<br>Ethnicity: Japanese	DM: 5.1%<br>HTN: 21%<br>SBP: N/A	Obesity: 33.8%	Proteinuria	Demographics, lifestyle, lipids	Obesity:<br>BMI ≥25 Kg/m^2	Overall: 1.45 (1.13-1.86)<br>Men: 1.60 (1.19-2.14)<br>Women: non-significant
Todd et al. 2015(6)	1998<br>(2004)	Finland	2,897	Age: 41<br>Male: 51%<br>Ethnicity: N/A	T1DM: 100%<br>HTN: N/A<br>SBP: N/A	BMI: 25.3 Kg/m^2	Macroalbuminuria	Demographics, diabetes duration	BMI: quintiles	U-shaped<br>Q5 vs Q2: 1.58 (1.06-2.36)
Chang et al. 2013(7)	1995-1996<br>(15 years)	USA	2,354	Age: 35<br>Male: 47%<br>African American: 41%	DM: 1.8%<br>HTN: 6%<br>SBP: 109 mmHg	BMI: 27 Kg/m^2<br>WC: 85.1 cm	Microalbuminuria: sex- and race-defined uACR ≥25 mg/g	Demographics, ACR	Obesity:<br>BMI ≥30 Kg/m^2	Overall: 2.2 (1.3-3.8)<br>Significant with adjustments of HTN, DM
Bello et al. 2007(8)	1991<br>(4.2 years)	Netherlands	6,894	Age: 49<br>Male: 50%<br>Caucasian: 96%	DM: N/A<br>HTN: N/A<br>SBP: 128 mmHg	BMI: stable weight (median-25.4 Kg/m^2)	Change in UAE: continuous	Demographics, medication	Weight change: per 1 Kg	β (p-value): 0.77 (0.004)
Amini et al. 2007(9)	1999<br>(5 years)	Iran	505	Age: 57.3<br>Male: 47%<br>Ethnicity: N/A	T2DM: 100%<br>HTN: N/A<br>SBP: 130 mmHg	BMI: 29.2 Kg/m^2	Microalbuminuria: urine albumin excretion of 30-300 mg/24h	Univariate analysis	BMI	Non-significant
Cross-sectional studies										
Cederholm et al. 2004(2)	2002	Sweden	40,172	Age: 61<br>Male: 54%<br>Ethnicity: N/A	DM: 100%<br>HTN: 73%<br>SBP: 140 mmHg	BMI: 28 Kg/m^2	Microalbuminuria: urine albumin 20-200 µg/min	Demographics, lifestyle, BP, DM duration, HbA1c	BMI:<br>per 1-Kg/m^2	T1DM: 3.18 (1.46-6.91)<br>T2DM: 8.07 (4.64-14.0)
Todd et al. 2015(6)	N/A	USA, UK , Finland	Cases: 2,347; control: 2,900	Age: cases (45), controls (41)<br>Male: 49%<br>Ethnicity: N/A	T1DM: 45%<br>HTN: N/A<br>SBP: N/A	BMI: cases (25.7 Kg/m^2), controls (25.7 Kg/m^2)	Macroalbuminuria	Demographics, DM duration	BMI	Overall: 1.28 (1.16-1.40)
Zheng et al. 2011(10)	2007.9-2008.11	China	2,080	Age: 51<br>Male: 39%	DM: 2%<br>HTN: 41.3%	BMI: 26.6 Kg/m^2	Microalbuminuria	Demographics, SBP, TC, HDL	BMI	Overall: 1.34 (1.06-1.69)

				Ethnicity: Chinese	SBP: 130.8 mmHg					
Zheng et al. 2010(11)	2007.9-2008.11	China	2,080	Age: 51<br>Male: 39%<br>Ethnicity: Chinese	DM: 2%<br>HTN: 41.3%<br>SBP: 130.8 mmHg	BMI: 26.6 Kg/m^2	Microalbuminuria	Multivariable analysis with stepwise selection	BMI	Men: not included<br>Women: 1.40 (1.13-1.74)
Liu et al. 2015(12)	2012.6-2013.2	China	944	Age: 61<br>Male: 44%<br>Ethnicity: Chinese	DM: 15.3%<br>HTN: 100%<br>SBP: 147 mmHg	BMI: 24.5 Kg/m^2<br>WC: 87.1 cm	Microalbuminuria	Age, SBP, glucose	BMI	Overall: 1.61 (1.22-2.10)
Tseng et al. 2005(13)	N/A	N/A	569	Age: 64<br>Male: 50%<br>Ethnicity: N/A	DM: 100%<br>HTN: N/A<br>SBP: 136 mmHg	BMI: 25 Kg/m^2<br>WC: 89 cm<br>WHtR: 0.56<br>WHR: 0.95	Microalbuminuria	N/A	BMI	Men: non-significant<br>Women: 1.40 (0.99-2.00)
Kaze et al. 2015(14)	2013.3-2013.4	Cameroon	500	Age: 45<br>Male: 53%<br>Ethnicity: N/A	DM: 2.8%<br>HTN: 12.2%<br>SBP: 132 mmHg	BMI: 27.1 Kg/m^2	Proteinuria	Age, sex	BMI	per 5-Kg/m^2: non-significant<br>per 1-SD:<br>Men: non-significant<br>Women: 1.42 (1.20-1.68)
Sato et al. 2013(15)	2008	Japan	212,251	Age: 65<br>Male: 40%<br>Ethnicity: Japanese	DM: N/A<br>HTN: N/A<br>SBP: N/A	BMI (median): 23.6 Kg/m^2 (men), 22.4 (women)	Proteinuria	Demographics, lifestyle, lipids, BP, eGFR, WC	BMI: cut-offs (11 categories)	Male: U-shaped<br>Female: U-shaped
Ramirez et al. 2002(16)	1999.1-2000.12	Singapore	189,117	Age: 36<br>Male: 53%<br>Chinese: 77.1%	DM: 2.1%<br>HTN: 6.6%<br>SBP: N/A	BMI ≥30 Kg/m^2: 5.7%	Proteinuria	Demographics , prior disease, BP, family history	BMI: cut-offs (18, 23, 25, 27.5, 30 Kg/m^2)	U-shaped<br>≥30 vs (18, 23): 2.5 (2.2-3.0)
Kim et al. 2013(17)	2008.11-2009.7	Korea	40,153	Age: 59<br>Male: 49%<br>Ethnicity: Korean	DM: 17.6%<br>HTN: 10%<br>SBP: 130 mmHg	BMI: 24.6 Kg/m^2	Albuminuria: uACR ≥30 mg/g in dipstick urinalysis	Demographics, lifestyle, DM, BP, medication	BMI: cut-offs (18.5, 23, 25 Kg/m^2)	Obesity vs normal weight: 1.47 (1.32-1.63)
Thoenes et al. 2009(18)	2005-2006	26 countries	20,868	Age: 62.4<br>Male: 52.3%<br>Ethnicity: N/A	DM: 27.5%<br>HTN: N/A<br>SBP: 149 mmHg	BMI: 28.9 Kg/m^2<br>WHR: 0.95<br>WC: 99.4 cm	Microalbuminuria: 30-150 mg/L in dipstick urinalysis	Multivariable analysis with stepwise selection	BMI: cut-offs (25, 28, 30, 35 Kg/m^2)	Non-significant
Kim et al. 2017(19)	2011-2014	Korea	19,331	Age: 46<br>Male: 45%<br>Ethnicity: Korean	DM: 10.2%<br>HTN: 29%<br>SBP: 117 mmHg	BMI: 23.7 Kg/m^2<br>WC: 80.7 cm	Albuminuria	Age, lifestyle, BP, DM duration, HbA1c	BMI: cut-offs (18.5, 23, 25 Kg/m^2)	≥25 vs [18.5, 23):<br>Men: 1.58 (1.21-2.07)<br>Women: 1.40 (1.08-1.81)
Lin et al. 2014(20)	2009.9-2012.6	China	10,384	Age: 53<br>Male: 43%<br>Ethnicity: Chinese	DM: N/A<br>HTN: N/A<br>SBP: 133 mmHg	BMI: 23.1 Kg/m^2	Microalbuminuria, macroalbuminuria, albuminuria	Demographics, HTN, DM, hyperuricemia	Obesity:<br>BMI ≥30 Kg/m^2	Microalbuminuria: 1.36 (1.10-1.69)<br>Macroalbuminuria: 1.59 (1.01-2.51)<br>Albuminuria: 1.39 (1.14-1.70)
Ren et. al 2016(21)	2011.6-2011.11	China	8,600	Age: 56<br>Male: 29%<br>Ethnicity: Chinese	DM: N/A<br>HTN: N/A<br>SBP: 126 mmHg	BMI: 23.6 Kg/m^2<br>WC: 81.7 cm	Albuminuria	Demographics, lifestyle, lipids, SBP, HbA1c, HOMA-IR	BMI: quartiles	Q4 vs Q1: 1.65 (1.26-2.18)
									Body fat content: quartiles	Non-significant
Kang et al. 2014(22)	2011	Korea	6,045	Age: 73<br>Male: 38%<br>Ethnicity: Korean	DM: 46.8%<br>HTN: 90.7%<br>SBP: 127 mmHg	BMI: 24.6 Kg/m^2	Albuminuria	Demographics, lifestyle, HTN, DM	BMI:<br>quartiles	Non-significant
Noh et al. 2017(23)	2010-2012	Korea	3,841	Age: approximate 43<br>Male: 45%<br>Ethnicity: Korean	DM: N/A<br>HTN: N/A<br>SBP: approximate 120 mmHg	BMI: 23.4 Kg/m^2<br>WC: 80.1 cm<br>WHtR: 0.49	Albuminuria	Demographics, lifestyle	BMI:<br>sex-specific quartiles	Male: non-significant<br>Female:<br>Q4 vs Q2: 2.40 (1.24-4.68)<br>Significant with adjustments of mediators
Dittmann	1997.10-	Germany	3,749	Age: median (men	DM: 8.5%	BMI (median):	Microalbuminuria:	Demographics,	BMI:	Non-significant

et al. 2013(24)	2001.3			[52], women [49])<br>Male: 51.3<br>Ethnicity: Caucasian	HTN: 52.6%<br>SBP: N/A	men (27.4 Kg/m^2), women (26.2 Kg/m^2)<br>women (0.50)	uACR ≥2.5 mg/mmol (men) or ≥3.5 mg/mmol (women)	T2DM, HTN	sex-specific quartiles	
Seo et al. 2016(25)	2013	Korea	3,257	Age: 50<br>Male: 44<br>Ethnicity: Korean	DM: 0<br>HTN: 0<br>SBP: 115 mmHg	BMI: 23.7 Kg/m^2<br>WC: 80.4 cm	Microalbuminuria	Demographics, BP, lipids, HbA1c, WC	BMI: cut-offs (18.5, 23, 25, 30 Kg/m^2)	Significant in underweight group vs normal weight
Du et al. 2014(26)	2010.1-2010.5	China	2,889	Age: 54<br>Male: 39%<br>Ethnicity: Chinese	DM: 100%<br>HTN: 50.3%<br>SBP: 131 mmHg	BMI: 24.8 Kg/m^2<br>WHR: 0.89	Albuminuria: uACR ≥17 mg/g (men) or ≥25 mg/g (women)	Demographics, lifestyle, HTN, blood glucose, lipids, uric acid	BMI: cut-offs (24, 28 Kg/m^2)	[24,28) vs <24: non-significant<br>≥28 vs <24: 1.61 (1.22-2.13)
Konta et al. 2006(27)	2004	Japan	2,321	Age: 64<br>Male: 45%<br>Ethnicity: Japanese	DM: 8.7%<br>HTN: 57%<br>SBP: 134 mmHg	BMI: 23.5 Kg/m^2	Microalbuminuria	Demographics, lifestyle, HTN, DM, urinary measurements	Obesity:<br>BMI ≥25 Kg/m^2	Non-significant
Wei et al. 2012(28)	2007.11-2009.3	China	1,187	Age: 41<br>Male: 42%<br>Ethnicity: Chinese	DM: 5%<br>HTN: 17%<br>SBP: 114 mmHg	BMI: 23.0 Kg/m^2	Albuminuria	Demographics, lipids, HTN, medications	BMI: cut-offs (23, 25, 30 Kg/m^2)	Obese II vs normal weight: 2.15 (1.00-2.20)
Belhatem et al. 2015(29)	2012.1-2013.12	France	Cases: 855;<br>Controls: 84	Age: cases (60), control (43);<br>Male: cases (60%), control (35%)<br>Ethnicity: N/A	DM: cases (100%)<br>HTN: N/A<br>SBP: cases (128 mmHg), control (123 mmHg)	BMI: cases (30.5 Kg/m^2), controls (38.1 Kg/m^2)	Micro- or macro-albuminuria: uACR ranged ≥2 times within 30-300 or >300 mg/g	Demographics, SBP, DM	BMI: cut-offs (25, 30, 40 Kg/m^2)	Morbid obese vs normal weight:<br>Microalbuminuria: 1.99 (1.35-2.98)<br>Macroalbuminuria: 2.33 (1.25-4.22)
Gojaseni et al. 2010(30)	2007.1-2007.12	Thailand	559	Age: 58<br>Male: 51%<br>Ethnicity: Thai	DM: 0<br>HTN: 100%<br>SBP: 141 mmHg	BMI: 26.1 Kg/m^2	Albuminuria: uACR >25 mg/g (women) or >17 mg/g (men)	Metabolic syndrome, BP, medication, abdominal obesity	Obesity:<br>BMI ≥30 Kg/m^2	Overall: 2.24 (1.33-3.76)
Al-Maskari et al. 2008(31)	2003.9-2004.5	United Arab Emirates	513	Age: 53<br>Male: 52%<br>Ethnicity: N/A	DM: 100%<br>HTN: 35%<br>SBP: N/A	BMI >30 Kg/m^2: 37.7%	Microalbuminuria: UAE of 20-200 µg/min or UPE rate of 30-300 µg/min	Demographics, T2DM, co-morbidity	Obesity:<br>BMI ≥25 Kg/m^2	0.61 (0.42-0.90)
Kohler et al. 2000(32)	1994.1.1-1996.12.31	USA	1,044	Age: 51.3<br>Male: 32%<br>Ethnicity: Black Americans	T2DM: 100%<br>HTN: N/A<br>SBP: N/A	BMI: 33.7 Kg/m^2	uACR	Demographics, HbA1c, lipids, DM duration , serum creatinine	BMI	β (SE): 0.0386 (0.0159)
Toto et al. 2010(33)	1994-1997	USA	652	Age: 60<br>Male: 61%<br>Ethnicity: African American	DM: N/A<br>HTN: N/A<br>SBP: 135 mmHg	BMI: 31.4 Kg/m^2	% of difference in geometric mean uPCRs/uACRs	Demographics, blood glucose, SBP	BMI:<br>per 2-Kg/m^2	uPCRs %: 3.5 (0.4-6.7)<br>uACRs %: 5.6 (1.6-8.9)
Foster et al. 2011(34)	2002.6-2005.3	USA	3,099	Age: 53<br>Male: 52%<br>Ethnicity: N/A	DM: 6.3%<br>HTN: 32%<br>SBP: 123 mmHg	BMI: 27.8 Kg/m^2<br>VAT: 1785 m^3<br>SAT: 2861.7 m^3<br>WC: 97.6 cm	Albuminuria: uACR >25 mg/g (women) or >17 mg/g (men)	Demographics, lifestyle, SBP, DM	BMI: per 1-SD	Men: 1.42 (1.20-1.68)<br>Women: Non-significant
									SAT:<br>per 1-SD	Men: 1.28 (1.08-1.52)<br>Women: 0.75 (0.59-0.97)
Kim et al. 2014(35)	2006.9-2010.12	Korea	1,154	Age: 56<br>Male: 68%<br>Ethnicity: Korean	DM: 16.7%<br>HTN: 40.6%<br>SBP: N/A	BMI: 24.4 Kg/m^2<br>VAT: 133 cm^2<br>SAT: 153 cm^2	Microalbuminuria	Demographics, lifestyle, DM, HTN, lipids,	SAT cross-sectional area: tertiles	Non-significant

								medication		
Genetic studies										
Todd et al. 2015(6)	N/A	USA, UK , Finland	Cases: 2,347; control: 2,900	Age: cases (45), controls (41)<br>Male: both 43%<br>Ethnicity: N/A	T1DM: 45%<br>HTN: N/A<br>SBP: N/A	BMI: cases (25.7 Kg/m^2), controls (25.7 Kg/m^2)	Macroalbuminuria	Age, sex, DM duration	BMI	Overall: 1.71 (1.25-2.24)
Central adiposity										
Cohort studies										
Retnakaran et al. 2006(36)	1977 (1991)	UK	4,031	Age: 53<br>Male: 60%<br>White Caucasian: 82%	DM: 100%<br>HTN: 45%<br>SBP: 135 mmHg	BMI: 27.7 Kg/m^2<br>WC: 96 cm	Microalbuminuria, macroalbuminuria	Demographics, lifestyle, BP, lipids, CVD	WC	Microalbuminuria: 1.10 (1.00-1.17)<br>Macroalbuminuria: 1.17 (1.06-1.29)
Bonnet et al. 2006(37)	6 years	France	2,738	Age: 47<br>Male: 54%<br>Ethnicity: N/A	DM: 0<br>HTN: 60%<br>SBP: 129 mmHg	BMI: 24.7 Kg/m^2<br>WC: 83.5 cm	Elevated albuminuria: UAE ≥20 mg/L or dipstick positive	Demographics, HTN, medication, blood glucose	WC	Men: 1.09 (1.00-1.48)<br>Women: 1.22 (1.00-1.63)
Boer et al. 2007(38)	5.8 years	USA	1,279	Age: 30<br>Men: 53%<br>White: 97%	DM: N/A<br>HTN: N/A<br>SBP: 117 mmHg	BMI: 25.8 Kg/m^2<br>BMI: 82.9 cm	Microalbuminuria: AER ≥30 mg/24h on two consecutive measures	Demographics, lifestyle, DM duration, HbA1c	WC	Overall: 1.34 (1.07-1.68)<br>Men: non-significant<br>Women: 1.65 (1.12-2.42)
Rossi et al. 2009(4)	N/A	Italy	1,019	Age: 63<br>Male: 64%<br>Ethnicity: N/A	DM: 100%<br>HTN: 62%<br>SBP: N/A	BMI: 29.2 Kg/m^2<br>WC: 101.3 cm	Progression of UAE: doubling in ACR from baseline to follow up	Multivariable analysis with stepwise selection	WC	Overall: 1..14 (1.00-1.32)
Lin et al. 2012(3)	2004.10 (2009.7)	Taiwan	2,350	Age: 57<br>Male: 49%<br>Ethnicity: Chinese	DM: 12.1%<br>HTN: N/A<br>SBP: 136 mmHg	BMI: 24.3 Kg/m^2<br>WC: 81.4 cm	Albuminuria: uACR≥30 mg/g	Demographics, lifestyle, BP, FPG, eGFR, BMI	Abdominal obesity: WC ≥90 cm (men) or ≥ 80 cm (women)	Overall: 2.12 (1.01-4.44)<br>Men: non-significant<br>Women: 3.13 (1.06-9.22)
Odagiri et al. 2014(39)	2008 (2.7 years)	Japan	4,841	Age: median (42)<br>Male: 76%<br>Ethnicity: Japanese	DM: 3.4%<br>HTN: 8.8%<br>SBP: 115 mmHg	BMI: 22 Kg/m^2	Proteinuria	Demographics, lifestyle, HTN, lipids, DM, HbA1c, eGFR	WHtR: quartiles	Q4 vs Q1: 2.68 (1.01, 7.10)<br>Non-significant with adjustment of BMI
Cross-sectional studies										
Tseng et al. 2005(13)	N/A	N/A	569	Age: 64<br>Male: 50%<br>Ethnicity: N/A	DM: 100%<br>HTN: N/A<br>SBP: 136 mmHg	BMI: 25 Kg/m^2<br>WC: 89 cm<br>WHtR: 0.56<br>WHR: 0.95	Microalbuminuria	N/A	WHR: per 0.1-unit	Men: non-significant<br>Women: 1.32 (0.97-1.80)
									WC	Men: non-significant<br>Women: 1.33 (1.03-1.71)
									WHtR: per 0.1-unit	Men: non-significant<br>Women: 1.64 (1.13-2.40)
Du et al. 2014(26)	2010.1-2010.5	Suzhou, China	2,889	Age: 54<br>Male: 39%<br>Ethnicity: Chinese	DM: 100%<br>HTN: 50.3%<br>SBP: 131 mmHg	BMI: 24.8 Kg/m^2<br>WHR: 0.89	Albuminuria: uACR ≥17 mg/g (men) or ≥25 mg/g (women)	Demographics, lifestyle, HTN, blood glucose, lipids	WHR: ≥0.9 (men) or ≥ 0.85 (women)	Overall: 1.31 (1.08-1.60)
Wang et al. 2016(40)	2008.3-2010.2	China	1,709	Age: 61<br>Male: 42%<br>Ethnicity: Chinese	T2DM: 100%<br>SBP: 149 mmHg	BMI: 26.2 Kg/m^2<br>WC: 86.7 cm<br>HC: 95.3 cm	Increased albuminuria: uACR≥10.18 mg/g	Demographics, lifestyle, family history	WHR: Sex-specific quartiles	Each quartile increment:<br>Overall: 1.23 (1.12-1.35)<br>Positive after adjustments of mediators and BMI
Foster et al. 2011(34)	2002.6-2005.3	USA	3,099	Age: 53<br>Male: 52%<br>Ethnicity: N/A	DM: 6.3%<br>HTN: 32%<br>SBP: 123 mmHg	BMI: 27.8 Kg/m^2<br>VAT: 1785 m^3<br>SAT: 2861.7 m^3	Albuminuria: uACR >25 (women) or >17 mg/g (men)	Demographics, lifestyle, HTN, DM	WC: per 1-SD	Men: 1.36 (1.14-1.63)<br>Women: non-significant

						WC: 97.6 cm			VAT:<br>per 1-SD	Men: 1.34 (1.12-1.60)<br>Women: non-significant
Chang et al. 2006(41)	2004.1-2004.12	Korea	1,321	Age: 45<br>Male: 100%<br>Ethnicity: Korean	DM: 0<br>HTN: 0<br>SBP: 112 mmHg	BMI: 23.8 Kg/m^2<br>WC: 83 cm	Microalbuminuria	Demographics, lifestyle, BP, glucose, lipids, WC	WC	Overall: 1.79 (1.10-2.83)
Rossi et al. 2010(4)	N/A	Italy	1,019	Age: 63<br>Male: 64%<br>Ethnicity: N/A	DM: 100%<br>HTN: 62%<br>SBP: N/A	BMI: 29.2 Kg/m^2<br>WC: 101.3 cm	Progression of UAE: doubling in ACR from baseline to follow-up	Multivariable analysis with stepwise selection	WC	Overall: 1.14 (1.00-1.32)
Liu et al. 2015(12)	2012.6-2013.2	China	944	Age: 61<br>Male: 44%<br>Ethnicity: Chinese	DM: 15.3%<br>HTN: 100%<br>SBP: 147 mmHg	BMI: 24.5 Kg/m^2<br>WC: 87.1 cm	Microalbuminuria	Age, SBP, FPG	WC	Non-significant
Bohm et al. 2007(42)	N/A	26 countries	21,050	Age: 62<br>Male: 52%	DM: 4.9%<br>HTN: 100%<br>SBP: 149 mmHg	BMI: 28.9 Kg/m^2<br>WHR: 0.95	Microalbuminuria: dipstick urinalysis	Multivariable analysis with stepwise selection	WC:<br>high vs normal	Overall: 1.13 (1.04-1.24)
Thoenes et al. 2009(18)	2005-2006	26 countries	20,868	Age: 62.4<br>Male: 52.3%<br>Ethnicity: N/A	DM: 27.5%<br>SBP: 149 mmHg	BMI: 28.9 Kg/m^2<br>WHR: 0.95<br>WC: 99.4 cm	Microalbuminuria: albumin excretion of 30, 80, or 150 mg/L in dipstick urinalysis	Multivariable analysis with stepwise selection	WC: ≥102 cm (men) or ≥ 88 cm (women)	Overall: 1.13 (1.04-1.23)
Kim et al. 2017(19)	2011-2014	Korea	19,331	Age: 46<br>Male: 45%<br>Ethnicity: Korean	DM: 10.2%<br>HTN: 29%<br>SBP: 117 mmHg	BMI: 23.7 Kg/m^2<br>WC: 80.7 cm	Albuminuria	Demographics, lifestyle, SBP, HbA1c, lipids	WC: Sex-specific quartiles	Q4 vs Q2:<br>Men: 2.17 (1.57-2.98)<br>Women: 1.79 (1.33-2.40)
Zhang et al. 2008(43)	N/A	China	13,925	Age: 40<br>Male: 54%<br>Ethnicity: Chinese	DM: 6.3%<br>HTN: 30%<br>SBP: N/A	Abdominal obesity: 38.5%	Albuminuria: uACR >25 (women) or >17 mg/g (men)	Demographics, lifestyle, medication, HTN, DM, lipids	Abdominal obesity: WC ≥90 cm (men) or ≥ 80 cm (women)	Overall: 1.40 (1.18-1.65)
Ren et. al 2016(21)	2011.6-2011.11	China	8,600	Age: 56<br>Male: 29%<br>Ethnicity: Chinese	DM: N/A<br>HTN: N/A<br>SBP: 126 mmHg	BMI: 23.6 Kg/m^2<br>WC: 81.7 cm	Albuminuria	Demographics, lifestyle, lipids, HbA1c, HOMA-IR, SBP	WC: quartiles	Q4 vs Q1: 1.73 (1.29-2.32)
Sarathy et al. 2016(44)	1999-2010	USA	6,918	Age: 30<br>Male: 46%<br>Non-Hispanic white: 47%	DM: 2.0%<br>HTN: 12%<br>SBP: 115 mmHg	BMI: 28.2 Kg/m^2<br>Abdominal obesity: 43.3%	Albuminuria	Demographics, lifestyle, lipids, HbA1c, SBP	Abdominal obesity: WC ≥102 cm (men) or ≥ 88 cm (women)	Non-Hispanic Whites: non-significant<br>Non-Hispanic Blacks: non-significant<br>Mexican-Americans: 3.0 (1.7-5.4)
Noh et al. 2017(23)	2010-2012	Korea	3,841	Age: approximate 43<br>Male: 45%<br>Ethnicity: Korean	DM: N/A<br>HTN: N/A<br>SBP: 120 mmHg	BMI: 23.4 Kg/m^2<br>WC: 80.1 cm<br>WHtR: 0.49	Albuminuria	Demographics, lfestyle	WC: sex-specific quartiles	Male: non-significant<br>Female:<br>Q4 vs Q2: 2.29 (1.17-4.49) (significant with adjustments of mediators)
									WHtR: sex-specific quartiles	Male: non-significant<br>Female:<br>Q4 vs Q2: 2.12 (1.07-4.19) (significant with adjustments of mediators)
Dittmann et al. 2013(24)	1997.10-2001.3	Germany	3,749	Age: median (men [52], women [49])<br>Male: 51.3<br>Ethnicity: Caucasian	DM: 8.5%<br>HTN: 52.6%<br>SBP: N/A	WC (median): men (95.5 cm), women (82.5 cm)<br>WHtR (median): men (0.55), women (0.50)	Microalbuminuria: uACR ≥2.5 mg/mmol (men) or ≥3.5 mg/mmol (women)	Age, sex, T2DM, HTN	WC: sex-specific quartiles	Q1 vs Q3: 1.48 (1.06-2.06)<br>Q5 vs Q3: 1.52 (1.16-2.00)
									WHtR: sex-specific quartiles	Q5 vs Q3: 1.33 (1.03-1.74)

Seo et al. 2016(25)	2013	Korea	3,257	Age: 50<br>Male: 44<br>Ethnicity: Korean	DM: 0<br>HTN: 0<br>SBP: 115 mmHg	BMI: 23.7 Kg/m^2<br>WC: 80.4 cm	Microalbuminuria	Demographics, glucose, HbA1c, BP, lipids, BMI	Abdominal obesity: WC ≥90 cm (men) or ≥ 80 cm (women)	Non-significant
Nguyen et al. 2008(45)	1999-2004	USA	2,515	Age: 15<br>Male: 53%<br>Non-Hispanic white: 26.8%	DM: 0.4%<br>HTN: 2.9%<br>SBP: N/A	Abdominal obesity: 20%	Microalbuminuria	Demographics	Abdominal obesity: WC ≥90% or >88 cm (women) and >102cm (men)	Non-significant
Lin et al. 2012(3)	2004.10	Taiwan	2,350	Age: 57<br>Male: 49%<br>Ethnicity: Chinese	DM: 12.1%<br>HTN: N/A<br>SBP: 136 mmHg	BMI: 24.3 Kg/m^2<br>WC: 81.4 cm	Albuminuria	Demographics, lifestyle, BP, CRP, glucose, eGFR, BMI	Abdominal obesity: WC ≥90 cm (men) or 80 cm (women)	Overall: 1.73 (1.04-2.85)<br>Men: non-significant<br>Women: 2.35 (1.10-5.01)
Gojaseni et al. 2010(30)	2007.1-2007.12	Thailand	559	Age: 58<br>Male: 51%<br>Ethnicity: Thai	DM: 0<br>HTN: 100%<br>SBP: 141 mmHg	BMI: 26.1 Kg/m^2	Albuminuria: uACR >25 mg/g (women) or >17 mg/g (men)	Metabolic syndrome, BP, medication, obesity	Abdominal obesity: WC ≥90 cm (men) or ≥ 80 cm (women)	Non-significant
Kim et al. 2014(35)	2006.9-2010.12	Korea	1,154	Age: 56<br>Male: 68%<br>Ethnicity: Korean	DM: 16.7%<br>HTN: 40.6%<br>SBP: N/A	BMI: 24.4 Kg/m^2<br>VAT: 133 cm^2<br>SAT: 153 cm^2	Microalbuminuria	Demographics, lifestyle, DM, HTN, lipids, medication	VAT cross-sectional area: tertiles	Overall:<br>T3 vs T1: 1.96 (1.12-3.43)
General +central adiposity										
Cross-sectional studies										
Pinto-Sietsma et al. 2003(46)	N/A	Netherlands	7,587	Age: 48<br>Male: 51%<br>Caucasian: 97%	DM: N/A<br>HTN: 30%<br>SBP: 128 mmHg	BMI: 25.9 Kg/m^2<br>WHR: 0.88	Microalbuminuria	Demographics, lifestyle, SBP, medication, lipids, glucose	BMI (25, 30 Kg/m^2) and WHR (0.9 [men], 0.8 [women])	Obese central fat vs lean peripheral fat: 1.7 (1.19-2.35)

* ORs or HRs of per 1-unit higher adiposity were converted to per 5-Kg/m^2 higher BMI and per 10-cm higher waist circumference for comparisons, which approximately equals ORs/HRs of per 1-SD higher adiposity in UK Biobank, unless otherwise stated.

Proteinuria is defined as grade ≥1+ in dipstick urinalysis; microalbuminuria is defined as uACR between 30-300 mg/g; macroalbuminuria is defined as uACR≥300 mg/g, and albuminuria is defined as uACR≥30 mg/g unless otherwise stated.

OR: odds ratio, HR: hazard ratio, DM: diabetes mellitus, HTN: hypertension, SBP: systolic blood pressure, WHR: waist-to-hip ratio, uACR: urinary albumin to creatinine ratio, HbA1c: haemoglobin A1c, BMI: body mass index, WC: waist circumference, eGFR: estimated glomerular filtration rate, VAT: visceral adipose tissue, SAT: subcutaneous adipose tissue, CVD: cardiovascular disease, BP: blood pressure, uPCR: urinary protein to creatinine ratio, T2DM: type 2 diabetes

References

1. Jang CM, Hyun YY, Lee KB, Kim H. The association between underweight and the development of albuminuria is different between sexes in relatively healthy Korean subjects. Nephrology Dialysis Transplantation. 2014 Nov;29(11):2106-13.
2. Cederholm J, Eliasson B, Nilsson PM, Weiss L, Gudbjornsdottir S. Microalbuminuria and risk factors in type 1 and type 2 diabetic patients. Diabetes Res Clin Pract. 2005 Mar;67(3):258-66.
3. Lin WY, Pi-Sunyer FX, Liu CS, Li CI, Davidson LE, Li TC, et al. Central obesity and albuminuria: both cross-sectional and longitudinal studies in Chinese. PLoS One. 2012;7(12):e47960.
4. Rossi MCE, Nicolucci A, Pellegrini F, Comaschi M, Ceriello A, Cucinotta D, et al. Obesity and changes in urine albumin/creatinine ratio in patients with type 2 diabetes: The DEMAND Study: Nutrition, Metabolism and Cardiovascular Diseases. Nutr Metab Cardiovasc Dis. 2010 Feb;20(2):110-6.
5. Tozawa M, Iseki K, Iseki C, Oshiro S, Ikemiya Y, Takishita S. Influence of smoking and obesity on the development of proteinuria. Kidney Int. 2002 Sep;62(3):956-62.
6. Todd JN, Dahlstrom EH, Salem RM, Sandholm N, Forsblom C, McKnight AJ, et al. Genetic evidence for a causal role of obesity in diabetic kidney disease. Diabetes. 2015 Dec;64(12):4238-46.
7. Chang A, Van Horn L, Jacobs Jr DR, Liu K, Muntner P, Newsome B, et al. Lifestyle-related factors, obesity, and incident microalbuminuria: The CARDIA (Coronary Artery Risk Development in Young Adults) study. Am J Kidney Dis. 2013 Aug;62(2):267-75.
8. Bello AK, de Zeeuw D, El Nahas M, Brantsma AH, Bakker SJL, de Jong PE, et al. Impact of weight change on albuminuria in the general population. Nephrol Dial Transplant. 2007 Jun;22(6):1619-27.
9. Amini M, Safaei H, Aminorroaya A. The incidence of microalbuminuria and its associated risk factors in type 2 diabetic patients in isfahan, Iran. Rev Diabet Stud. 2007 Winter; 4(4): 242–248.
10. Zheng J, Ye P, Wang X, Xiao WK, Wu HM. [The relationship between obesity and microalbuminuria among general population in Beijing area]. [Chinese]. Chung Hua Nei Ko Tsa Chih Chinese Journal of Internal Medicine. 2011;50(5):388-92.
11. Zheng J, Ye P, Wang F, Xiao WK, Wu HM. [Prevalence of microalbuminuria and its related factors among different genders in general population of Beijing]. [Chinese]. Chung Hua Liu Hsing Ping Hsueh Tsa Chih Chinese Journal of Epidemiology. 2010;31(11):1250-4.
12. Liu X, Liu Y, Chen Y, Li Y, Shao X, Liang Y, et al. Body mass index (BMI) is associated with microalbuminuria in chinese hypertensive patients. Int J Environ Res Public Health. 2015 Feb; 12(2): 1998–2008.
13. Tseng C-H. Waist-to-Height Ratio Is Independently and Better Associated With Urinary Albumin Excretion Rate Than Waist Circumference or Waist-to-Hip Ratio in Chinese Adult Type 2 Diabetic Women but Not Men. Diabetes Care. 2005 Sep;28(9):2249-51.
14. Kaze FF, Halle MP, Mopa HT, Ashuntantang G, Fouda H, Ngogang J, et al. Prevalence and risk factors of chronic kidney disease in urban adult Cameroonians according to three common estimators of the glomerular filtration rate: a cross-sectional study. BMC Nephrol. 2015; 16: 96.
15. Sato Y, Fujimoto S, Konta T, Iseki K, Moriyama T, Yamagata K, et al. U-shaped association between body mass index and proteinuria in a large Japanese general population sample. Clin Exp Nephrol. 2014 Feb;18(1):75-86.
16. Ramirez SP, McClellan W, Port FK, Hsu SI. Risk factors for proteinuria in a large, multiracial, southeast Asian population. J Am Soc Nephrol. 2002 Jul;13(7):1907-17.
17. Kim YS, Kim HS, Oh HY, Lee MK, Kim CH, Kim YS, et al. Prevalence of microalbuminuria and associated risk factors among adult Korean hypertensive patients in a primary care setting. Hypertens Res. 2013 Sep;36(9):807-23.
18. Thoenes M, Reil JC, Khan BV, Bramlage P, Volpe M, Kirch W, et al. Abdominal obesity is associated with microalbuminuria and an elevated cardiovascular risk profile in patients with hypertension. Vasc Health Risk Manag. 2009;5(4):577-85.
19. Kim YJ, Hwang SD, Oh TJ, Kim KM, Jang HC, Kimm H, et al. Association Between Obesity and Chronic Kidney Disease, Defined by Both Glomerular Filtration Rate and Albuminuria, in Korean Adults. Metab Syndr Relat Disord. 2017 Oct;15(8):416-422.

20. Lin B, Shao L, Luo Q, Ou-yang L, Zhou F, Du B, et al. Prevalence of chronic kidney disease and its association with metabolic diseases: a cross-sectional survey in Zhejiang province, Eastern China. BMC Nephrol. 2014; 15: 36.
21. Ren M, Sun K, Li F, Qi YQ, Lin DZ, Li N, et al. Association between obesity measures and albuminuria: A population-based study. J Diabetes Complications. 2016 Apr;30(3):451-6.
22. Kang YU, Bae EH, Ma SK, Kim SW. Determinants and burden of chronic kidney disease in a high-risk population in Korea: results from a cross-sectional study. Korean J Intern Med. 2016 Sep;31(5):920-9.
23. Noh HM, Kim UY, Park YS, Song YR, Oh HY, Park KH, et al. Sex differences in the association between obesity and albuminuria among Korean adults: a cross-sectional study using the Korea National Health and Nutrition Examination Survey data. Clin Exp Nephrol. 2017 Feb;21(1):27-34.
24. Dittmann K, Hannemann A, Wallaschofski H, Rettig R, Stracke S, Volzke H, et al. U-shaped association between central body fat and the urinary albumin-to-creatinine ratio and microalbuminuria. BMC Nephrol. 2013 Apr;14:87.
25. Seo WJ, Lee GM, Hwang JH, Lee MN, Kang HC. Association between Body Mass Index, Waist Circumference and Prevalence of Microalbuminuria in Korean Adults of Age 30 Years and Older without Diabetes, Hypertension, Renal Failure, or Overt Proteinuria: The 2013 Korean National Health and Nutrition Examination Survey. Korean J Fam Med. 2016 Jan;37(1):57-63.
26. Du N, Peng H, Chao X, Zhang Q, Tian H, Li H. Interaction of obesity and central obesity on elevated urinary albumin-to-creatinine ratio. PLoS One. 2014 Jun 3;9(6):e98926.
27. Konta T, Hao Z, Abiko H, Ishikawa M, Takahashi T, Ikeda A, et al. Prevalence and risk factor analysis of microalbuminuria in Japanese general population: the Takahata study. Kidney Int. 2006 Aug;70(4):751-6.
28. Wei X, Li Z, Chen W, Mao H, Li Z, Dong X, et al. Prevalence and risk factors of chronic kidney disease in first-degree relatives of chronic kidney disease patients in Southern China. Nephrology. 2012;17(2):123-30.
29. Belhatem N, Mohammedi K, Rouzet F, Matallah N, Al Baloshi A, Travert F, et al. Impact of morbid obesity on the kidney function of patients with type 2 diabetes. Diabetes Res Clin Pract. 2015 Apr;108(1):143-9.
30. Gojaseni P, Phaopha A, Chailimpamontree W, Pajareya T, Chittinandana A. Prevalence and risk factors of microalbuminuria in Thai nondiabetic hypertensive patients. Vasc Health Risk Manag. 2010 Mar 24;6:157-65.
31. Al-Maskari F, El-Sadig M, Obineche E. Prevalence and determinants of microalbuminuria among diabetic patients in the United Arab Emirates. BMC Nephrol. 2008 Jan 29;9:1.
32. Kohler KA, McClellan WM, Ziemer DC, Kleinbaum DG, Boring JR. Risk factors for microalbuminuria in black americans with newly diagnosed type 2 diabetes. Am J Kidney Dis. 2000 Nov;36(5):903-13.
33. Toto RD, Greene T, Hebert LA, Hiremath L, Lea JP, Lewis JB, et al. Relationship between body mass index and proteinuria in hypertensive nephrosclerosis: results from the African American Study of Kidney Disease and Hypertension (AASK) cohort. Am J Kidney Dis. 2010 Nov;56(5):896-906.
34. Foster MC, Hwang SJ, Massaro JM, Hoffmann U, DeBoer IH, Robins SJ, et al. Association of subcutaneous and visceral adiposity with albuminuria: the Framingham Heart Study. Obesity. 2011 Jun;19(6):1284-9.
35. Kim H, Kim HJ, Shin N, Han M, Park H, Kim M, et al. Visceral obesity is associated with microalbuminuria in nondiabetic Asians. Hypertens Res. 2014 Jul;37(7):679-84.
36. Retnakaran R, Cull CA, Thorne KI, Adler AI, Holman RR. Risk factors for renal dysfunction in type 2 diabetes: U.K. Prospective Diabetes Study 74. Diabetes. 2006 Jun;55(6):1832-9.
37. Bonnet F, Marre M, Halimi JM, Stengel B, Lange C, Laville M, et al. Waist circumference and the metabolic syndrome predict the development of elevated albuminuria in non-diabetic subjects: the DESIR Study. J Hypertens. 2006 Jun;24(6):1157-63.
38. de Boer IH, Sibley SD, Kestenbaum B, Sampson JN, Young B, Cleary PA, et al. Central obesity, incident microalbuminuria, and change in creatinine clearance in the epidemiology of diabetes interventions and complications study. J Am Soc Nephrol. 2007 Jan;18(1):235-43.
39. Odagiri K, Mizuta I, Yamamoto M, Miyazaki Y, Watanabe H, Uehara A. Waist to height ratio is an independent predictor for the incidence of chronic kidney disease. PLoS One. 2014 Feb 12;9(2):e88873.

40. Wang Z, Ding L, Huang X, Sun W, Lin L, Huang Y, et al. Abdominal adiposity contributes to adverse glycemic control and albuminuria in Chinese type 2 diabetic patients: A cross-sectional study. J Diabetes. 2017 Mar;9(3):285-295.
41. Chang Y, Yoo T, Ryu S, Huh BY, Cho BL, Sung E, et al. Abdominal obesity, systolic blood pressure, and microalbuminuria in normotensive and euglycemic Korean men. Int J Obes. 2006 May;30(5):800-4.
42. Bohm M, Thoenes M, Danchin N, Reil JC, Volpe M. Overview of the i-SEARCH Global Study : Cardiovascular Risk Factors and Microalbuminuria in Hypertensive Individuals. High Blood Press Cardiovasc Prev. 2008 Oct;15(4):217-24.
43. Zhang L, Zhang P, Wang F, Zuo L, Zhou Y, Shi Y, et al. Prevalence and factors associated with CKD: a population study from Beijing. Am J Kidney Dis. 2008 Mar;51(3):373-84.
44. Sarathy H, Henriquez G, Abramowitz MK, Kramer H, Rosas SE, Johns T, et al. Abdominal obesity, race and chronic kidney disease in young adults: Results from NHANES 1999-2010. PLoS One. 2016 May 25;11(5):e0153588.
45. Nguyen S, McCulloch C, Brakeman P, Portale A, Hsu CY. Being overweight modifies the association between cardiovascular risk factors and microalbuminuria in adolescents. Pediatrics. 2008 Jan;121(1):37-45.
46. Pinto-Sietsma SJ, Navis G, Janssen WM, de Zeeuw D, Gans RO, de Jong PE, et al. A central body fat distribution is related to renal function impairment, even in lean subjects. Am J Kidney Dis. 2003 Apr;41(4):733-41.

Table S3: Baseline characteristics of UK Biobank by fifths of waist-to-hip ratio

Characteristics	I	II	III	IV	V	All (n=408,527)
**Adiposity**						
Waist-to-hip ratio	0.78 (0.06)	0.83 (0.06)	0.87 (0.06)	0.91 (0.06)	0.97 (0.06)	0.87 (0.09)
Waist-to-height ratio	0.46 (0.05)	0.50 (0.05)	0.53 (0.05)	0.57 (0.05)	0.62 (0.05)	0.53 (0.07)
Waist circumference (cm)	78 (10)	85 (10)	90 (10)	95 (10)	103 (10)	90 (13)
Trunk fat (%)	25.8 (7.2)	29.0 (7.1)	31.2 (7.1)	33.3 (7.1)	35.7 (7.1)	31.0 (7.9)
BMI (kg/m²)	24.1 (4.1)	25.8 (4.1)	27.2 (4.1)	28.8 (4.1)	31.0 (4.1)	27.4 (4.7)
Height-adjusted weight (kg)	68.8 (13.6)	73.7 (13.5)	77.5 (13.5)	82.1 (13.5)	88.1 (13.6)	78.0 (15.0)
Hip circumference (cm)	100 (9)	101 (9)	103 (9)	105 (9)	107 (9)	103 (9)
Body fat (%)	26.4 (7.9)	29.2 (7.8)	31.4 (7.8)	33.3 (7.8)	35.8 (7.9)	31.2 (8.5)
**Socio-demographics**						
Age, years	53.9 (8.3)	55.4 (8.2)	56.4 (8.0)	57.3 (7.8)	58.1 (7.6)	56.2 (8.1)
White	77,800 (95.9%)	77,762 (95.5%)	77,534 (94.7%)	76,401 (94.0%)	76,400 (92.3%)	385,897 (94.5%)
College or University degree	34,241 (40.2%)	29,644 (35.6%)	26,586 (32.4%)	23,015 (28.8%)	19,669 (24.7%)	133,155 (32.6%)
Townsend deprivation score	-2.4 (-3.8, -0.0)	-2.4 (-3.8, 0.1)	-2.3 (-3.7, 0.3)	-2.1 (-3.6, 0.6)	-1.7 (-3.4, 1.3)	-2.2 (-3.7, 0.5)
**Lifestyle**						
Current smoker	7,129 (8.0%)	8,037 (9.5%)	8,546 (10.4%)	8,870 (11.2%)	10,056 (12.9%)	42,638 (10.4%)
Daily drinker	16,287 (20.9%)	17,393 (21.6%)	17,212 (20.9%)	16,730 (20.2%)	15,647 (18.3%)	83,269 (20.4%)
Physical activity (MET-hr/wk)	35.9 (34.5)	33.5 (34.4)	32.2 (34.6)	30.5 (34.9)	28.4 (35.5)	32.2 (33.3)
Urinary sodium-to-creatinine ratio (mmol/mmol) $	9.5 (6.4, 13.5)	9.6 (6.5, 13.5)	9.6 (6.5, 13.6)	9.6 (6.4, 13.7)	9.7 (6.4, 14.0)	9.6 (6.4, 13.7)
**Health status**						
Diabetes †	1,319 (1.4%)	2,060 (2.1%)	3,224 (3.1%)	5,405 (5.2%)	11,629 (10.9%)	23,637 (5.8%)
HbA1c (%)	6.7 (6.0, 7.7)	6.7 (6.0, 7.4)	6.7 (6.1, 7.4)	6.7 (6.2, 7.4)	6.9 (6.3, 7.7)	6.8 (6.2, 7.6)
Duration of diabetes, years	6.0 (1.0, 20.0)	4.0 (1.0, 10.0)	3.0 (0.0, 9.0)	3.0 (0.0, 8.0)	4.0 (0.0, 8.0)	4.0 (0.0, 9.0)
Prediabetes †	824 (1.0%)	1,293 (1.4%)	2,080 (2.2%)	3,155 (3.2%)	5,155 (5.1%)	12,507 (3.1%)
HbA1c (%)	6.1 (6.0, 6.2)	6.1 (6.0, 6.2)	6.1 (6.0, 6.2)	6.1 (6.0, 6.2)	6.1 (6.1, 6.3)	6.1 (6.0, 6.2)
Prediabetes †	79,534 (97.6%)	78,331 (96.5%)	76,618 (94.7%)	72,527 (91.5%)	65,373 (83.6%)	372,383 (91.2%)
HbA1c (%)	5.3 (5.1, 5.5)	5.3 (5.1, 5.5)	5.3 (5.1, 5.5)	5.4 (5.2, 5.6)	5.4 (5.2, 5.6)	5.3 (5.1, 5.5)
Any vascular diseases ‡	2,164 (3.0%)	3,125 (3.9%)	4,052 (4.7%)	5,301 (5.8%)	7,676 (7.9%)	22,318 (5.5%)
Systolic blood pressure (mmHg)	134 (18)	137 (17)	138 (17)	139 (17)	140 (18)	138 (19)
Diastolic blood pressure (mmHg)	79 (10)	81 (10)	83 (10)	84 (10)	85 (10)	82 (10)
eGFR (mL/min/1.73m²) §	91.5 (12.0)	91.3 (11.9)	91.2 (11.9)	91.0 (11.9)	90.9 (12.0)	91.2 (13.2)
**Outcome**						
Urinary albumin-to-creatinine ratio (mg/mmol) $	1.0 (0.6, 1.9)	1.0 (0.6, 1.9)	1.0 (0.6, 1.9)	1.0 (0.6, 2.0)	1.1 (0.7, 2.4)	1.0 (0.6, 2.0)

Age-adjusted mean (SD), N (%), or median (Q1, Q3) shown by fifths of waist-to-hip ratio and arithmetic mean (SD), N (%) or median (Q1, Q3) shown overall, unless otherwise stated. BMI: body mass index, HbA1c: glycosylated haemoglobin, eGFR: estimated glomerular filtration rate. $ Mean (SD) or median (Q1, Q3) among those with detectable values. † Diabetes is defined as self-reported diabetes or HbA1c>=6.5%, prediabetes is defined as HbA1c between 5.7-<6.5%, no diabetes is defined as HbA1c<5.7%. ‡ Any vascular diseases include self-reported heart attack, angina, or stroke. § eGFR was calculated from the Chronic Kidney Disease Epidemiology Collaboration (CKD-EPI) creatinine equation. Exclusion criteria: participants with self-reported cancer, chronic obstructive pulmonary disease or liver failure/cirrhosis; with missing values of adiposity measures, blood pressure, HbA1c, or urinary albumin-to-creatinine ratio.

Table S4: Baseline characteristics of UK Biobank by fifths of BMI

Characteristics	I	II	III	IV	V	All (n=408,527)
**Adiposity**						
Waist-to-hip ratio	0.82 (0.08)	0.85 (0.08)	0.87 (0.08)	0.89 (0.08)	0.92 (0.08)	0.87 (0.09)
Waist-to-height ratio	0.46 (0.04)	0.50 (0.04)	0.53 (0.04)	0.56 (0.04)	0.63 (0.04)	0.53 (0.07)
Waist circumference (cm)	78 (9)	84 (9)	89 (9)	94 (9)	106 (9)	90 (13)
Trunk fat (%)	23.0 (5.7)	28.0 (5.7)	31.1 (5.7)	34.1 (5.7)	38.9 (5.7)	31.0 (7.9)
BMI (kg/m²)	21.9 (1.9)	24.7 (1.9)	26.7 (1.9)	29.1 (1.9)	34.6 (1.9)	27.4 (4.7)
Height-adjusted weight (kg)	62.7 (9.1)	70.5 (9.1)	76.1 (9.1)	82.9 (9.1)	98.0 (9.1)	78.0 (15.0)
Hip circumference (cm)	95 (6)	99 (6)	102 (6)	106 (6)	115 (6)	103 (9)
Body fat (%)	23.8 (6.8)	28.3 (6.7)	31.1 (6.7)	34.0 (6.8)	38.8 (6.7)	31.2 (8.5)
**Socio-demographics**						
Age, years	55.1 (8.3)	56.1 (8.2)	56.6 (8.1)	56.9 (8.0)	56.5 (7.9)	56.2 (8.1)
White	77,243 (94.9%)	77,529 (94.9%)	77,439 (94.7%)	77,034 (94.1%)	76,652 (93.7%)	385,897 (94.5%)
College or University degree	34,693 (41.4%)	29,922 (36.3%)	25,976 (31.8%)	22,884 (28.1%)	19,680 (23.9%)	133,155 (32.6%)
Townsend deprivation score	-2.2 (-3.7, 0.4)	-2.4 (-3.8, -0.0)	-2.3 (-3.7, 0.1)	-2.2 (-3.6, 0.5)	-1.6 (-3.4, 1.4)	-2.2 (-3.7, 0.5)
**Lifestyle**						
Current smoker	10,318 (12.2%)	8,328 (10.1%)	8,130 (10.0%)	8,053 (10.0%)	7,809 (9.6%)	42,638 (10.4%)
Daily drinker	19,515 (24.2%)	18,587 (22.6%)	17,407 (20.9%)	15,771 (18.8%)	11,989 (14.4%)	83,269 (20.4%)
Physical activity (MET-hr/wk)	35.0 (34.3)	34.0 (34.3)	32.6 (34.5)	31.2 (34.8)	27.7 (35.4)	32.2 (33.3)
Urinary sodium-to-creatinine ratio (mmol/mmol) $	9.8 (6.6, 13.9)	9.6 (6.5, 13.7)	9.5 (6.5, 13.6)	9.5 (6.4, 13.5)	9.5 (6.2, 13.6)	9.6 (6.4, 13.7)
**Health status**						
Diabetes †	1,650 (1.7%)	2,246 (2.2%)	3,188 (3.1%)	5,142 (4.9%)	11,411 (11.4%)	23,637 (5.8%)
HbA1c (%)	6.7 (6.0, 7.7)	6.7 (6.1, 7.4)	6.7 (6.1, 7.5)	6.8 (6.2, 7.6)	6.8 (6.3, 7.6)	6.8 (6.2, 7.6)
Duration of diabetes, years	5.0 (1.0, 16.0)	4.0 (1.0, 11.0)	4.0 (0.0, 10.0)	4.0 (0.0, 9.0)	3.0 (0.0, 8.0)	4.0 (0.0, 9.0)
Prediabetes †	1,003 (1.1%)	1,440 (1.5%)	1,985 (2.1%)	3,007 (3.1%)	5,072 (5.4%)	12,507 (3.1%)
HbA1c (%)	6.1 (6.0, 6.2)	6.1 (6.0, 6.2)	6.1 (6.0, 6.2)	6.1 (6.1, 6.2)	6.1 (6.1, 6.3)	6.1 (6.0, 6.2)
Prediabetes †	79,044 (97.2%)	78,040 (96.3%)	76,516 (94.9%)	73,566 (91.9%)	65,217 (82.7%)	372,383 (91.2%)
HbA1c (%)	5.3 (5.1, 5.5)	5.3 (5.1, 5.5)	5.3 (5.1, 5.5)	5.4 (5.1, 5.6)	5.4 (5.2, 5.6)	5.3 (5.1, 5.5)
Any vascular diseases ‡	2,563 (3.2%)	3,236 (3.8%)	4,117 (4.6%)	5,122 (5.7%)	7,280 (8.5%)	22,318 (5.5%)
Systolic blood pressure (mmHg)	133 (17)	136 (17)	138 (17)	140 (17)	142 (17)	138 (19)
Diastolic blood pressure (mmHg)	78 (10)	81 (10)	82 (10)	84 (10)	86 (10)	82 (10)
eGFR (mL/min/1.73m²) §	93.2 (11.8)	91.6 (11.8)	90.9 (11.8)	90.2 (11.8)	90.0 (11.8)	91.2 (13.2)
**Outcome**						
Urinary albumin-to-creatinine ratio (mg/mmol) $	1.1 (0.7, 2.1)	1.0 (0.6, 1.9)	1.0 (0.6, 1.9)	1.0 (0.6, 1.9)	1.1 (0.7, 2.2)	1.0 (0.6, 2.0)

Age-adjusted mean (SD), N (%), or median (Q1, Q3) shown by fifths of BMI and arithmetic mean (SD), N (%) or median (Q1, Q3) shown overall, unless otherwise stated. BMI: body mass index, HbA1c: glycosylated haemoglobin, eGFR: estimated glomerular filtration rate. $ Mean (SD) or median (Q1, Q3) among those with detectable values. † Diabetes is defined as self-reported diabetes or HbA1c>=6.5%, prediabetes is defined as HbA1c between 5.7-<6.5%, no diabetes is defined as HbA1c<5.7%. ‡ Any vascular diseases include self-reported heart attack, angina, or stroke. § eGFR was calculated from the Chronic Kidney Disease Epidemiology Collaboration (CKD-EPI) creatinine equation. Exclusion criteria: participants with self-reported cancer, chronic obstructive pulmonary disease or liver failure/cirrhosis; with missing values of adiposity measures, blood pressure, HbA1c, or urinary albumin-to-creatinine ratio.

Table S5: Sex-specific mean waist-to-hip ratio and BMI by selected characteristics

	Men			Women		
**Characteristics**	**N**	**Waist-to-hip ratio**	**BMI(kg/m²)**	**N**	**Waist-to-hip ratio**	**BMI(kg/m²)**
**Age, years**						
35-49	46,334	0.92 (0.06)	27.6 (4.2)	53,902	0.80 (0.07)	26.4 (5.3)
50-59	61,913	0.93 (0.06)	27.9 (4.3)	75,997	0.82 (0.07)	27.1 (5.2)
60-74	82,139	0.95 (0.06)	27.9 (4.0)	88,242	0.83 (0.07)	27.3 (4.8)
**Ethnicity**						
White	179,611	0.94 (0.06)	27.8 (4.2)	206,286	0.82 (0.07)	26.9 (5.0)
Others	10,775	0.93 (0.06)	27.5 (4.5)	11,855	0.84 (0.07)	28.0 (6.1)
**Highest qualification completed $**						
College or University degree	64,727	0.92 (0.06)	27.0 (3.9)	68,428	0.80 (0.07)	26.1 (4.9)
A levels/GCSEs or equivalent	91,424	0.94 (0.06)	28.1 (4.2)	111,327	0.82 (0.07)	27.2 (5.1)
None of the above	34,235	0.96 (0.07)	28.6 (5.2)	38,386	0.84 (0.07)	28.2 (6.3)
**UK Region**						
London or South	57,608	0.93 (0.06)	27.4 (4.1)	69,378	0.81 (0.07)	26.5 (5.0)
Midlands	31,065	0.94 (0.06)	27.9 (4.2)	33,008	0.82 (0.07)	27.2 (5.1)
North	80,014	0.94 (0.06)	28.0 (4.2)	90,078	0.82 (0.07)	27.2 (5.1)
Wales	8,074	0.94 (0.06)	28.4 (4.3)	9,188	0.82 (0.07)	27.6 (5.3)
Scotland	13,625	0.93 (0.06)	27.8 (4.2)	16,489	0.81 (0.07)	27.0 (5.0)
**Townsend deprivation score (fifths) †**						
1 (Least deprived)	38,595	0.93 (0.06)	27.5 (3.8)	44,106	0.81 (0.07)	26.3 (4.5)
2	38,305	0.93 (0.06)	27.7 (4.0)	44,045	0.81 (0.07)	26.6 (4.7)
3	38,198	0.93 (0.06)	27.8 (4.1)	43,926	0.81 (0.07)	26.9 (4.9)
4	38,135	0.94 (0.06)	27.9 (4.3)	43,479	0.82 (0.07)	27.2 (5.2)
5 (Most deprived)	36,903	0.94 (0.07)	28.2 (4.8)	42,336	0.83 (0.07)	28.1 (5.8)
**Smoking status**						
Ever	95,511	0.94 (0.06)	28.1 (4.3)	86,602	0.82 (0.07)	27.1 (5.1)
Never	93,945	0.93 (0.06)	27.5 (4.1)	130,519	0.81 (0.07)	26.9 (5.1)
**Physical activity, MET-hr/wk ‡**						
Lower	87,094	0.94 (0.06)	28.0 (4.3)	97,834	0.82 (0.07)	27.4 (5.2)
Higher	88,448	0.93 (0.06)	27.4 (3.9)	99,148	0.81 (0.07)	26.3 (4.6)
**Urinary sodium-to-creatinine ratio (fifths)**						
M: (0, 5.4]; F: (0, 6.2]	38,040	0.94 (0.06)	28.0 (4.3)	43,503	0.82 (0.07)	27.4 (5.3)
M: (5.4, 7.6]; F: (6.2, 9.0]	38,041	0.93 (0.06)	27.7 (4.1)	43,504	0.82 (0.07)	27.0 (5.0)
M: (7.6, 10.0]; F: (9.0, 12.0]	38,041	0.93 (0.06)	27.7 (4.1)	43,503	0.81 (0.07)	26.9 (5.0)
M: (10.0, 13.3]; F: (12.0, 16.2]	38,041	0.93 (0.06)	27.8 (4.1)	43,504	0.82 (0.07)	26.9 (5.0)
M: >13.3; F: >16.2	38,040	0.94 (0.06)	27.8 (4.4)	43,503	0.82 (0.07)	26.9 (5.1)
**Diabetes status ¶**						
No diabetes	169,635	0.93 (0.06)	27.5 (3.9)	202,748	0.81 (0.07)	26.7 (4.8)
Prediabetes	6,058	0.97 (0.06)	30.3 (5.2)	6,449	0.86 (0.07)	31.0 (6.9)
Diabetes	14,693	0.98 (0.07)	31.1 (5.7)	8,944	0.88 (0.08)	32.0 (6.8)
**Any self-reported vascular diseases §**						
No	175,020	0.93 (0.06)	27.7 (4.1)	211,189	0.82 (0.07)	26.9 (5.0)
Yes	15,366	0.96 (0.07)	29.5 (6.0)	6,952	0.85 (0.07)	29.6 (7.3)

Arithmetic mean (SD) of adiposity measures for age, and age-adjusted mean (SD) of adiposity measures for the rest. BMI: body mass index, HbA1c: glycosylated haemoglobin, M: men, F: women. $ A levels/GCSEs or equivalent include A levels, AS levels, O levels, GCSEs, CSEs, NVQ, HND, HNC, other professional qualifications or equivalent. † Range of Townsend deprivation score in each fifth by sex: women (1 [-6.3, -3.9], 2 [-3.9, -2.8], 3 [-2.8, -1.3], 4 [-1.3, 1.2], 5 [1.2, 11]); men (1 [-6.3, -3.9], 2 [-3.9, -2.8], 3 [-2.8, -1.3], 4 [-1.3, 1.4], 5 [1.4, 11]). ‡ Physical activity was categoried according to sex-specific median (women: 20.28 MET-hr/wk, men: 22.50 MET-hr/wk). ¶ Diabetes is defined as self-reported diabetes or HbA1c>=6.5%, prediabetes is defined as HbA1c between 5.7-<6.5%, no diabetes is defined as HbA1c<5.7%. § Any vascular diseases include heart attack, angina, or stroke. Exclusion criteria: participants with self-reported cancer, chronic obstructive pulmonary disease or liver failure/cirrhosis; with missing values of adiposity measures, blood pressure, HbA1c, or urinary albumin-to-creatinine ratio.

Table S6: Within-person variability of adiposity measures during resurveys by sex

Adiposity	No of participants	Mean (SD) at baseline	Mean (SD) at resurvey (2012-13)	Regression dilution ratio (MacMahon's non-parametric method)	Regression dilution ratio (Rosner's regression method)
**Men**					
Central adiposity					
Waist-to-hip ratio	8,198	0.93 (0.06)	0.94 (0.06)	0.64	0.71
Waist-to-height ratio	8,195	0.54 (0.06)	0.55 (0.06)	0.83	0.81
Waist circumference (cm)	8,200	95 (11)	97 (11)	0.83	0.82
Trunk fat (%)	8,116	27.0 (6.6)	28.1 (6.6)	0.85	0.84
General adiposity					
BMI (kg/m^2)	8,190	27.3 (4.0)	27.4 (4.0)	0.94	0.92
Height-adjusted weight (kg)	8,190	84.2 (12.3)	84.3 (12.4)	0.94	0.92
Hip circumference (cm)	8,198	103 (7)	103 (8)	0.83	0.78
Body fat (%)	8,111	24.7 (5.7)	25.6 (5.7)	0.87	0.86
**Women**					
Central adiposity					
Waist-to-hip ratio	8,292	0.81 (0.07)	0.82 (0.06)	0.65	0.70
Waist-to-height ratio	8,289	0.51 (0.07)	0.52 (0.08)	0.88	0.80
Waist circumference (cm)	8,292	82 (12)	84 (12)	0.87	0.80
Trunk fat (%)	8,218	33.6 (7.6)	34.0 (7.6)	0.83	0.84
General adiposity					
BMI (kg/m^2)	8,290	26.4 (4.8)	26.5 (4.9)	0.94	0.91
Height-adjusted weight (kg)	8,290	69.8 (12.6)	69.7 (12.6)	0.93	0.92
Hip circumference (cm)	8,292	102 (10)	103 (10)	0.89	0.79
Body fat (%)	8,218	36.0 (6.7)	36.5 (6.7)	0.87	0.87

BMI: body mass index, HbA1c: glycosylated haemoglobin. Exclusion criteria: participants with self-reported cancer, chronic obstructive pulmonary disease or liver failure/cirrhosis; with missing values of adiposity measures, blood pressure, HbA1c, or urinary albumin-to-creatinine ratio.

Table S7a: Associations between incremental increase in central adiposity and a higher urinary albumin-to-creatinine ratio category

		Adiposity adjustment							
		BMI		Height-adjusted weight		Hip circumference		Body fat%	
**Exposures**	**Increment**	**OR (95% CI)**	χ^2	**OR (95% CI)**	χ^2	**OR (95% CI)**	χ^2	**OR (95% CI)**	χ^2
**Waist-to-hip ratio**									
Confounder-adjusted model	0.06	1.55 (1.53, 1.57)	3,683	1.55 (1.53, 1.57)	3,683	1.55 (1.53, 1.57)	3,683	1.55 (1.53, 1.57)	3,683
Adiposity-adjusted model		1.32 (1.30, 1.34)	1,297	1.32 (1.30, 1.34)	1,294	1.46 (1.44, 1.48)	2,618	1.39 (1.36, 1.41)	1,819
Mediator-adjusted model		1.24 (1.22, 1.26)	770	1.24 (1.22, 1.26)	753	1.32 (1.30, 1.34)	1,348	1.36 (1.34, 1.38)	1,738
**Waist-to-height ratio**									
Confounder-adjusted model	0.07	1.50 (1.48, 1.52)	5,598	1.50 (1.48, 1.52)	5,598	1.50 (1.48, 1.52)	5,598	1.50 (1.48, 1.52)	5,598
Adiposity-adjusted model		1.32 (1.30, 1.34)	1,269	1.31 (1.29, 1.32)	1,278	1.46 (1.44, 1.48)	3,323	1.44 (1.42, 1.46)	2,834
Mediator-adjusted model		1.24 (1.22, 1.26)	741	1.22 (1.20, 1.24)	688	1.31 (1.29, 1.32)	1,608	1.34 (1.32, 1.35)	2,655
**Waist circumference (cm)**									
Confounder-adjusted model	12.1	1.53 (1.51, 1.55)	5,560	1.53 (1.51, 1.55)	5,560	1.53 (1.51, 1.55)	5,560	1.53 (1.51, 1.55)	5,560
Adiposity-adjusted model		1.33 (1.31, 1.35)	1,257	1.32 (1.30, 1.34)	1,138	1.54 (1.52, 1.57)	3,474	1.47 (1.45, 1.49)	2,848
Mediator-adjusted model		1.26 (1.24, 1.28)	846	1.25 (1.23, 1.27)	726	1.37 (1.35, 1.40)	1,793	1.36 (1.35, 1.38)	2,712
**Trunk fat (%) †**									
Confounder-adjusted model	7.3	1.44 (1.42, 1.46)	2,878	1.44 (1.42, 1.46)	2,878	1.44 (1.42, 1.46)	2,878	1.44 (1.42, 1.46)	2,878
Adiposity-adjusted model		1.07 (1.05, 1.09)	67	1.06 (1.04, 1.07)	42	1.25 (1.23, 1.27)	794	-	-
Mediator-adjusted model		1.05 (1.04, 1.07)	40	1.04 (1.02, 1.06)	20	1.16 (1.14, 1.17)	322	1.27 (1.26, 1.29)	1,220

BMI: body mass index; uACR: urinary albumin-to-creatinine ratio; SBP: systolic blood pressure; DBP: diastolic blood pressure. Log ORs for a higher uACR category per incremental increase in adiposity are the inverse variance weighted averages of the sex-specific log ORs (calculated as the slopes of the inverse variance weighted regression through the log ORs of the top four adiposity categories). Confounder-adjusted model was adjusted for age, ethnicity, education, region, Townsend deprivation index, smoking, and physical activity at baseline. Adiposity-adjusted model was further adjusted for each of the adiposity measures (BMI, height-adjusted weight, hip circumference, and body fat [%]) at baseline. Mediator-model was further adjusted for diabetes status (diabetes, prediabetes, no diabetes), duration of diabetes, SBP, DBP, and self-reported vascular disease (heart attack, angina, or stroke). † Trunk fat (%) was not adjusted for body fat (%) due to high correlation between the two measures at baseline.

Table S7b: Associations between incremental increase in general adiposity and a higher urinary albumin-to-creatinine ratio category

		Adiposity adjustment							
		Waist-to-hip ratio		Waist-to-height ratio		Waist circumference		Trunk fat%	
Exposures	Increment	OR (95% CI)	χ^2	OR (95% CI)	χ^2	OR (95% CI)	χ^2	OR (95% CI)	χ^2
**BMI (kg/m²)**									
Confounder-adjusted model	5	1.47 (1.46, 1.49)	5,171	1.47 (1.46, 1.49)	5,171	1.47 (1.46, 1.49)	5,171	1.47 (1.46, 1.49)	5,171
Adiposity-adjusted model		1.35 (1.33, 1.37)	2,731	1.18 (1.17, 1.20)	478	1.20 (1.18, 1.21)	555	1.42 (1.40, 1.44)	2,750
Mediator-adjusted model		1.23 (1.22, 1.25)	1,278	1.12 (1.10, 1.13)	196	1.11 (1.09, 1.13)	185	1.32 (1.30, 1.33)	2,390
**Height-adjusted weight (kg)**									
Confounder-adjusted model	14	1.49 (1.48, 1.51)	5,461	1.49 (1.48, 1.51)	5,461	1.49 (1.48, 1.51)	5,461	1.49 (1.48, 1.51)	5,461
Adiposity-adjusted model		1.37 (1.35, 1.38)	2,902	1.21 (1.19, 1.23)	662	1.21 (1.19, 1.23)	579	1.45 (1.43, 1.47)	2,956
Mediator-adjusted model		1.25 (1.24, 1.27)	1,424	1.15 (1.13, 1.17)	330	1.13 (1.11, 1.14)	228	1.33 (1.32, 1.35)	2,611
**Hip circumference (cm)**									
Confounder-adjusted model	9.2	1.34 (1.32, 1.35)	2,745	1.34 (1.32, 1.35)	2,745	1.34 (1.32, 1.35)	2,745	1.34 (1.32, 1.35)	2,745
Adiposity-adjusted model		1.26 (1.25, 1.27)	1,660	1.06 (1.05, 1.08)	85	1.00 (0.99, 1.01)	0	1.22 (1.20, 1.23)	841
Mediator-adjusted model		1.18 (1.17, 1.19)	808	1.05 (1.04, 1.07)	56	1.00 (0.98, 1.01)	0	1.22 (1.21, 1.23)	1,202
**Body fat (%) †**									
Confounder-adjusted model	7.6	1.56 (1.53, 1.58)	3,495	1.56 (1.53, 1.58)	3,495	1.56 (1.53, 1.58)	3,495	1.56 (1.53, 1.58)	3,495
Adiposity-adjusted model		1.35 (1.33, 1.38)	1,490	1.09 (1.07, 1.11)	87	1.09 (1.07, 1.11)	88	-	-
Mediator-adjusted model		1.22 (1.20, 1.24)	619	1.05 (1.03, 1.07)	23	1.03 (1.01, 1.05)	12	1.34 (1.32, 1.37)	1,478

BMI: body mass index; uACR: urinary albumin-to-creatinine ratio; SBP: systolic blood pressure; DBP: diastolic blood pressure. Log ORs for a higher uACR category per incremental increase in adiposity are the inverse variance weighted averages of the sex-specific log ORs (calculated as the slopes of the inverse variance weighted regression through the log ORs of the top four adiposity categories). Confounder-adjusted model was adjusted for age, ethnicity, education, region, Townsend deprivation index, smoking, and physical activity at baseline. Adiposity-adjusted model was further adjusted for each of the adiposity measures (waist-to-hip ratio, waist-to-height ratio, waist circumference, and trunk fat [%]) at baseline. Mediator-model was further adjusted for diabetes status (diabetes, prediabetes, no diabetes), duration of diabetes, SBP, DBP, and self-reported vascular disease (heart attack, angina, or stroke). † Body fat (%) was not adjusted for trunk fat (%) due to high correlation between the two measures at baseline.

Figure S1: Flow chart of participants' selection, with reasons for exclusion

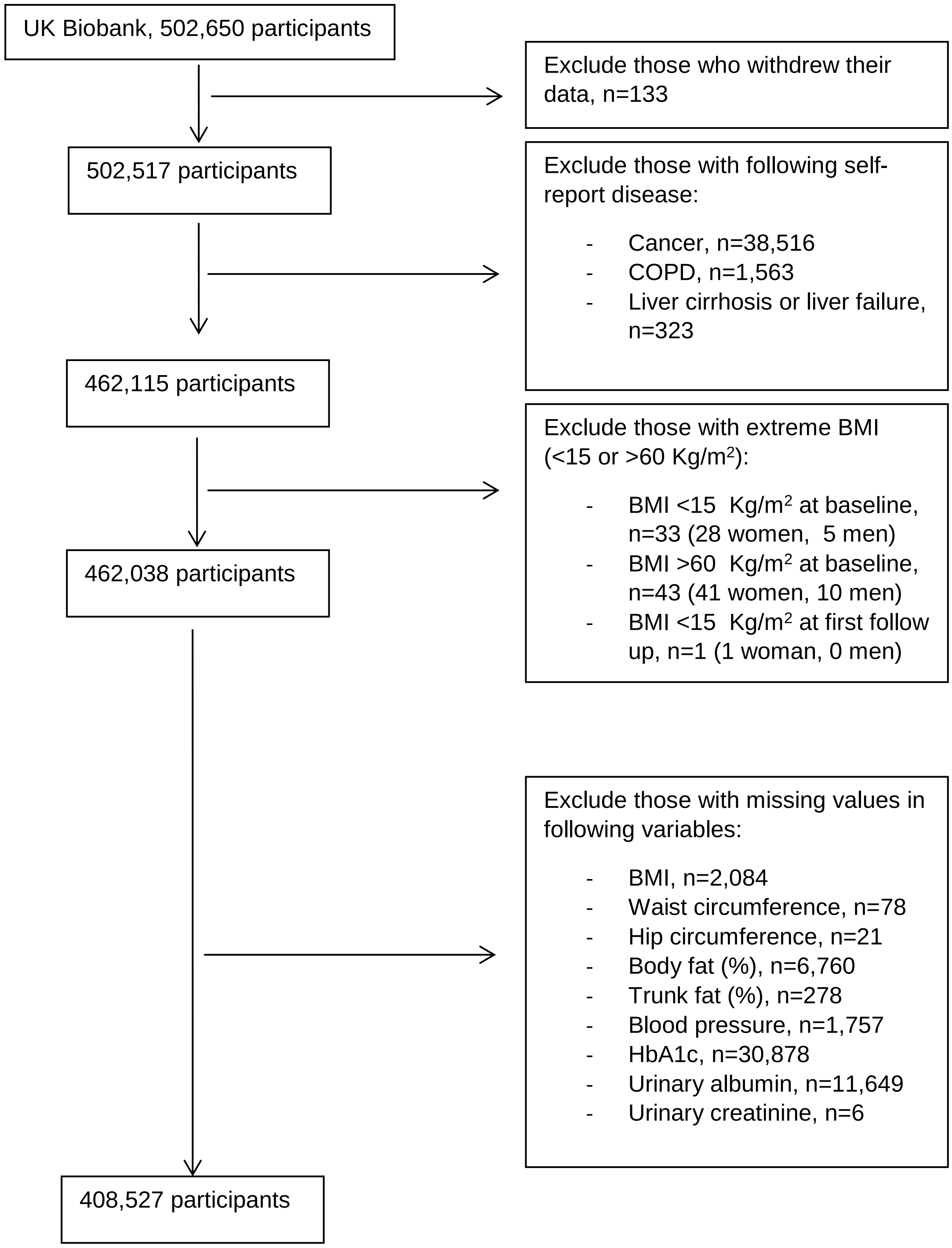

COPD: chronic obstructive pulmonary disease; BMI: body mass index; HbA1c: glycosylated haemoglobin.

Figure S2: Directed acyclic graph showing assumed associations between baseline measured adiposity, characteristics, and albuminuria

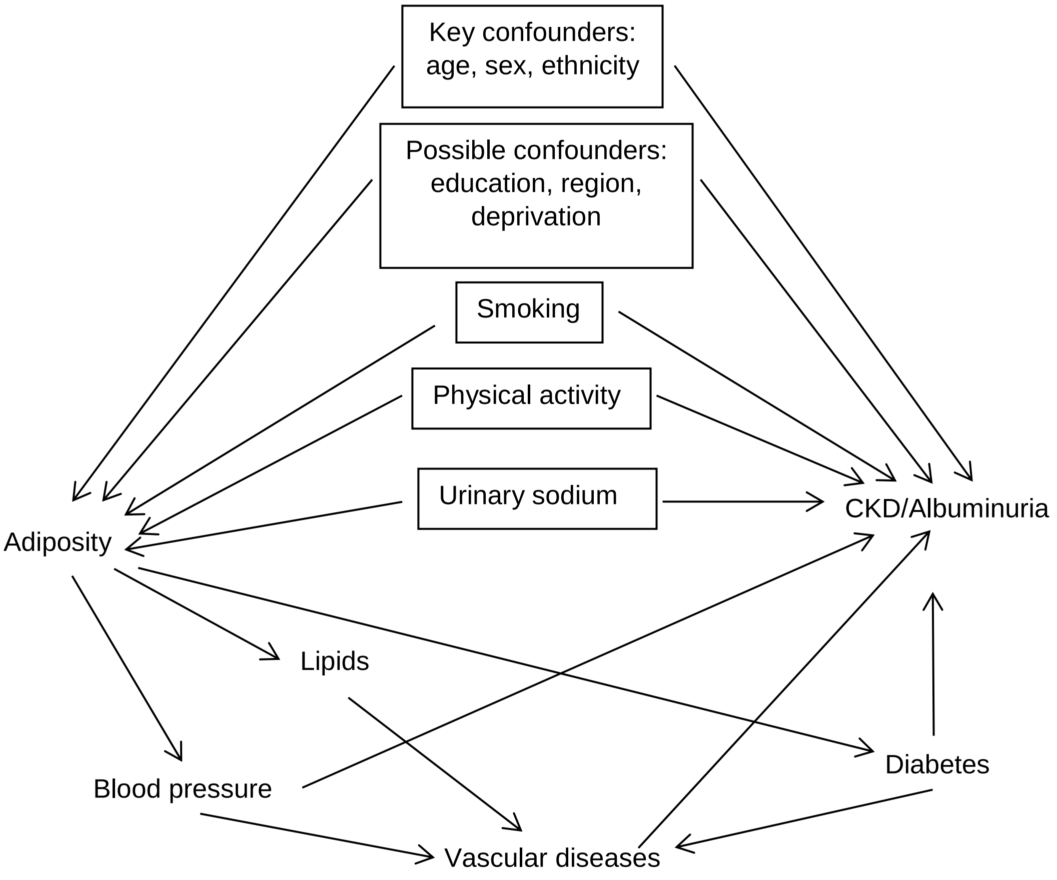

1. Exposures: measures of adiposity; outcome: albuminuria;
2. Key confounders (age, sex, and ethnicity) are chosen as they are correlated with the exposure and outcome but cannot be changed by exposure and outcome;
3. Possible confounders (region, education, and deprivation) are associated with the exposure and outcome and unlikely to be changed by exposure and outcome;
4. Smoking and physical activity are widely accepted to modify weight, whereas some uncertainties remained if these factors affect albuminuria risk;
5. Some studies also suggested the possible confounding effect of dietary sodium (measured by urinary sodium) in association between adiposity and kidney function;
6. Blood pressure, vascular diseases (heart attack, angina, or stroke), and possibly lipids are effect mediators which may be effected by adiposity and have an effect on albuminuria risk;
7. Arrows between confounders and effect mediators are omitted to simplify the causal diagram;
8. Alcohol is not included as there is no certain evidence of an association between alcohol and risk of albuminuria;
9. Though an indirect association between lipids and albuminuria is presented, trial evidence does not suggest ow-density lipoprotein cholesterol or high-density lipoprotein cholesterol has an effect on albuminuria risk (i.e. no direct causal pathway between lipids and albuminuria is assumed);
10. Variables enclosed by boxes are considered confounders and will be adjusted for during analyses (all models);
11. Analyses adjusting for diabetes, systolic blood pressure, and vascular diseases are conducted to assess the mediation effect of these factors; no adjustment is made for lipids as conditioning on vascular diseases closes the pathway between lipids and albuminuria.

Figure S3: Sex−specific associations between BMI and central adiposity measures

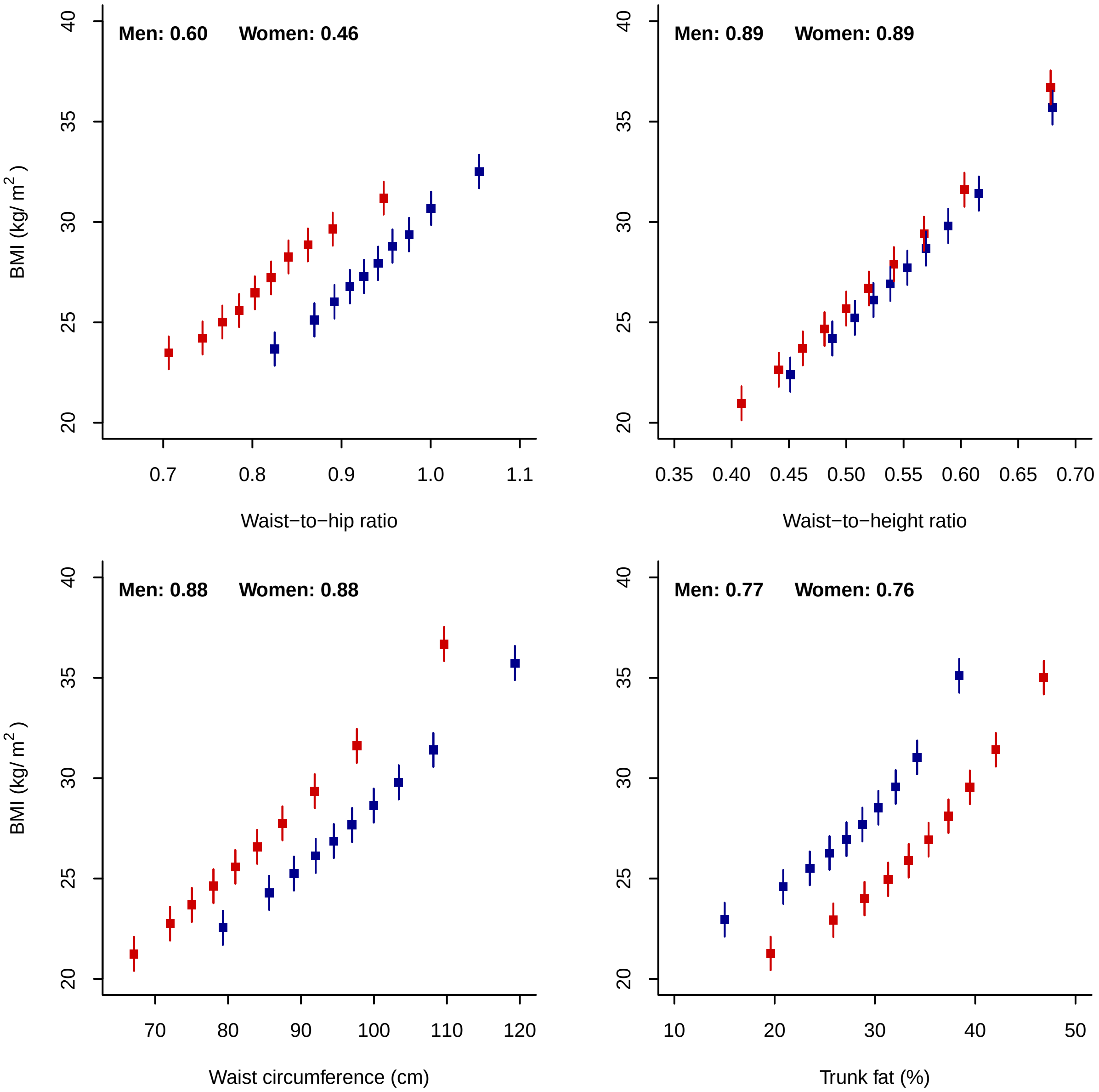

Red: women; blue: men.
BMI: body mass index, HbA1c: glycosylated haemoglobin.
Age−adjusted associations between adiposity measures in individuals without cancer, chronic obstructive pulmonary disease, or liver failure/cirrhosis, and without missing values in adiposity measures, blood pressure, HbA1c, or urinary creatinine are shown.
Age−adjusted Pearson correlation coefficients are shown on plots.

Figure S4: Associations between incremental increase in adiposity (waist−to−hip ratio and BMI) and a higher urinary albumin−to−creatinine ratio category overall and by sex

Adjustments	Increment	Odds Ratio (95% CI) per incremental increase in adiposity	χ^2
**Waist−to−hip ratio**			
Men			
Confounders	0.06	1.75 (1.71, 1.79)	2710
+ BMI		1.39 (1.36, 1.42)	789
+ Mediators		1.30 (1.27, 1.33)	501
Women			
Confounders	0.06	1.40 (1.38, 1.43)	1202
+ BMI		1.27 (1.24, 1.30)	540
+ Mediators		1.20 (1.17, 1.22)	298
All			
Confounders	0.06	1.55 (1.53, 1.57)	3683
+ BMI		1.32 (1.30, 1.34)	1297
+ Mediators		1.24 (1.22, 1.26)	770
**BMI**			
Men			
Confounders	5 kg/m^2	1.71 (1.68, 1.74)	3718
+ Waist−to−hip ratio		1.50 (1.47, 1.53)	1794
+ Mediators		1.33 (1.31, 1.36)	876
Women			
Confounders	5 kg/m^2	1.35 (1.33, 1.37)	1906
+ Waist−to−hip ratio		1.27 (1.25, 1.29)	1125
+ Mediators		1.18 (1.16, 1.20)	502
All			
Confounders	5 kg/m^2	1.47 (1.46, 1.49)	5171
+ Waist−to−hip ratio		1.35 (1.33, 1.37)	2731
+ Mediators		1.23 (1.22, 1.25)	1278

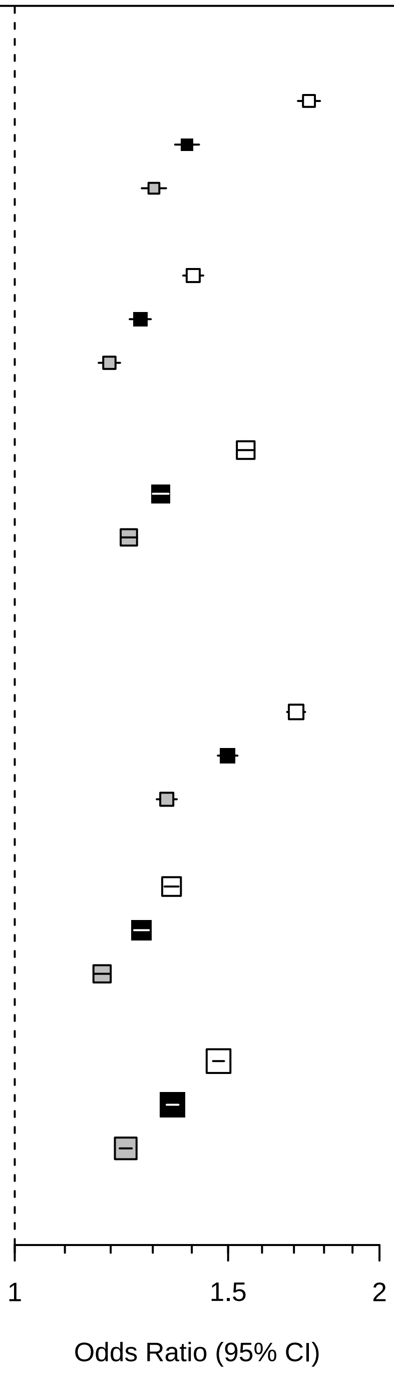

BMI: body mass index, uACR: urinary albumin−to−creatinine ratio,
SBP: systolic blood pressure, DBP: diastolic blood pressure, HbA1c: glycosylated haemoglobin.
Log ORs for a higher uACR category per incremental increase in adiposity overall are the inverse variance weighted averages of the sex−specific log ORs (calculated as the slopes of the inverse variance weighted regressions through the log ORs of the top four adiposity categories).
Confounders: age, ethnicity, education, region, Townsend deprivation index, smoking, physical activity at baseline.
Mediators: diabetes status (diabetes, prediabetes, no diabetes), duration of diabetes, SBP, DBP, and any self−reported vascular disease [heart attack, angina, or stroke]) at baseline.

Figure S5: Associations between incremental increase in adiposity (waist−to−hip ratio and BMI) and albuminuria (urinary albumin−to−creatinine ratio of 3 mg/mmol or greater) overall

Adjustments	Increment	Odds Ratio (95% CI) per incremental increase in adiposity	χ^2
Waist−to−hip ratio			
Confounders	0.06	1.62 (1.57, 1.67)	1023
+ BMI		1.43 (1.38, 1.47)	477
+ Mediators		1.27 (1.23, 1.31)	206
BMI			
Confounders	5 kg/m^2	1.47 (1.43, 1.50)	1157
+ Waist−to−hip ratio		1.31 (1.28, 1.34)	494
+ Mediators		1.12 (1.09, 1.15)	80

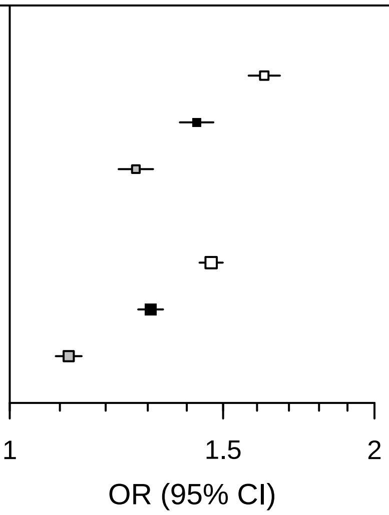

BMI: body mass index, SBP: systolic blood pressure, DBP: diastolic blood pressure, uACR: urinary albumin−to−creatinine ratio.
Log ORs for albuminuria (uACR of 3 mg/mmol or greater) per incremental increase in adiposity overall are the inverse variance weighted averages of the sex−specific log ORs (calculated as the slopes of the inverse variance weighted regressions through the log ORs of the top four adiposity categories).
Confounders: age, ethnicity, education, region, Townsend deprivation index, smoking, and physical activity at baseline.
Mediators: diabetes status (diabetes, prediabetes, no diabetes), duration of diabetes, SBP, DBP, and any self−reported vascular disease (heart attack, angina, or stroke) at baseline.

Figure S6: Associations between incremental increase in adiposity (other 4 adiposity measures) and a higher urinary albumin−to−creatinine ratio category by sex

Adjustments	Increment	Odds Ratio (95% CI) per incremental increase in adiposity	χ^2
**Men**			
Central adiposity			
Waist−to−height ratio			
Confounders	0.07	1.72 (1.69, 1.75)	3811
+ Height−adjusted weight		1.40 (1.37, 1.43)	767
Waist circumference			
Confounders	12.1cm	1.70 (1.67, 1.73)	3619
+ Hip circumference		1.75 (1.71, 1.79)	2409
General adiposity			
Height−adjusted weight			
Confounders	14kg	1.62 (1.60, 1.65)	3681
+ Waist−to−height ratio		1.28 (1.25, 1.31)	500
Hip circumference			
Confounders	9.2cm	1.55 (1.52, 1.58)	1939
+ Waist circumference		0.98 (0.95, 1.00)	4
**Women**			
Central adiposity			
Waist−to−height ratio			
Confounders	0.07	1.38 (1.36, 1.40)	2187
+ Height−adjusted weight		1.25 (1.23, 1.27)	564
Waist circumference			
Confounders	12.1cm	1.42 (1.40, 1.44)	2184
+ Hip circumference		1.41 (1.38, 1.44)	1270
General adiposity			
Height−adjusted weight			
Confounders	14kg	1.39 (1.37, 1.41)	1984
+ Waist−to−height ratio		1.16 (1.14, 1.18)	206
Hip circumference			
Confounders	9.2cm	1.25 (1.23, 1.27)	1125
+ Waist circumference		1.01 (0.99, 1.03)	2

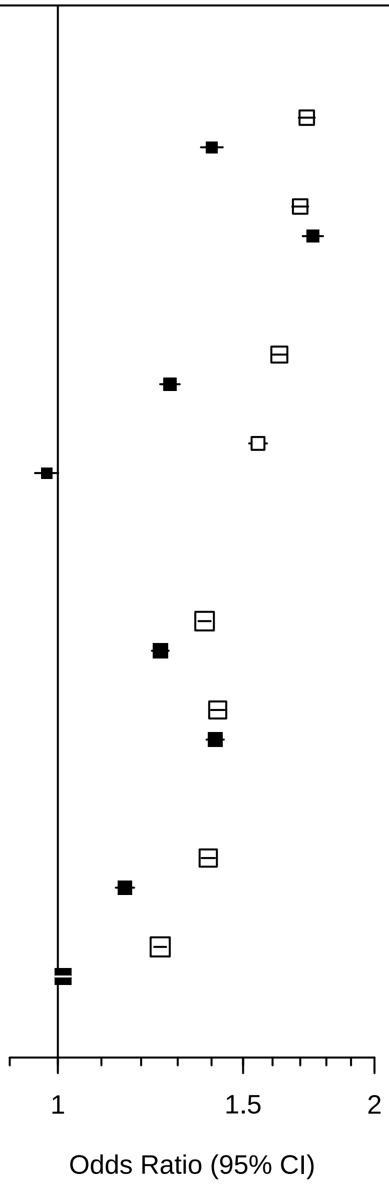

BMI: body mass index, uACR: urinary albumin−to−creatinine ratio, SBP: systolic blood pressure, DBP: diastolic blood pressure.
Log ORs for a higher uACR category per incremental increase in adiposity overall are the inverse variance weighted averages of the sex−specific log ORs (calculated as the slopes of the inverse variance weighted regressions through the log ORs of the top four adiposity categories).
Confounders: age, ethnicity, education, region, Townsend deprivation index, smoking, and physical activity at baseline.

Figure S7: Associations between incremental increase in adiposity (waist−to−hip ratio and BMI) and a higher urinary albumin−to−creatinine ratio category without adjustments for measurement error overall and by sex

Adjustments	Increment	Odds Ratio (95% CI) per incremental increase in adiposity	χ^2
**Waist−to−hip ratio**			
Men			
Confounders	0.06	1.42 (1.40, 1.44)	2710
+ BMI		1.23 (1.21, 1.25)	790
+ Mediators		1.18 (1.16, 1.20)	501
Women			
Confounders	0.06	1.23 (1.22, 1.24)	1222
+ BMI		1.16 (1.14, 1.17)	553
+ Mediators		1.12 (1.10, 1.13)	306
All			
Confounders	0.06	1.31 (1.30, 1.32)	3678
+ BMI		1.19 (1.18, 1.20)	1305
+ Mediators		1.14 (1.13, 1.15)	775
**BMI**			
Men			
Confounders	5 kg/m^2	1.63 (1.61, 1.66)	3718
+ Waist−to−hip ratio		1.45 (1.43, 1.48)	1796
+ Mediators		1.30 (1.28, 1.33)	878
Women			
Confounders	5 kg/m^2	1.32 (1.30, 1.33)	1910
+ Waist−to−hip ratio		1.25 (1.23, 1.27)	1129
+ Mediators		1.17 (1.15, 1.18)	505
All			
Confounders	5 kg/m^2	1.43 (1.41, 1.44)	5179
+ Waist−to−hip ratio		1.32 (1.30, 1.33)	2738
+ Mediators		1.21 (1.20, 1.23)	1283

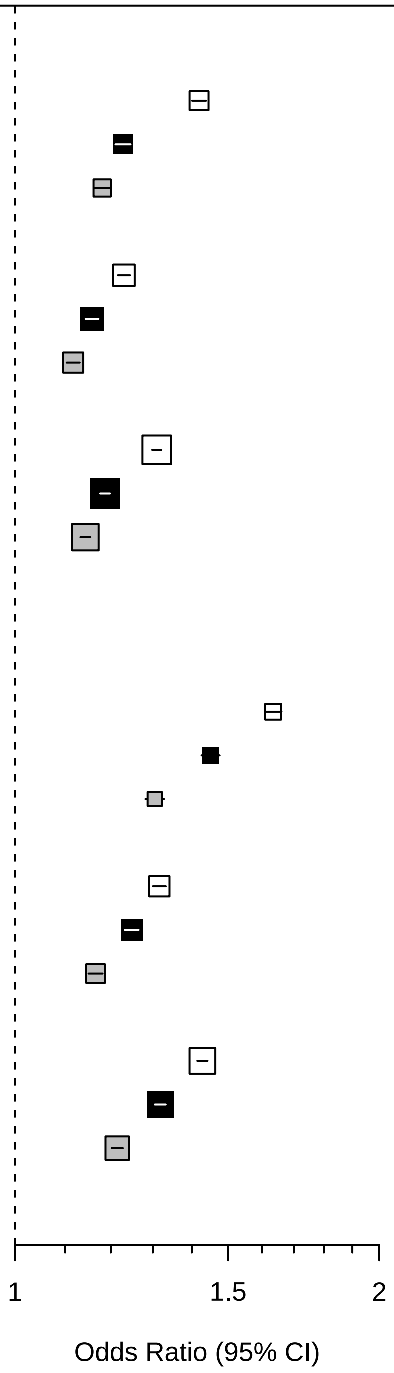

BMI: body mass index, uACR: urinary albumin−to−creatinine ratio,
SBP: systolic blood pressure, DBP: diastolic blood pressure, HbA1c: glycosylated haemoglobin.
Log ORs for a higher uACR category per incremental increase in adiposity overall are the inverse variance weighted averages of the sex−specific log ORs (calculated as the slopes of the inverse variance weighted regressions through the log ORs of the top four adiposity categories).
Confounders: age, ethnicity, education, region, Townsend deprivation index, smoking, physical activity at baseline.
Mediators: diabetes status (diabetes, prediabetes, no diabetes), duration of diabetes, SBP, DBP, and any self−reported vascular disease [heart attack, angina, or stroke]) at baseline.

Figure S8: Associations between adiposity (waist−to−hip adjusted for BMI and BMI adjusted for waist−to−hip ratio) and albuminuria by sex and by halves of urinary sodium−to−creatinine ratio

Men

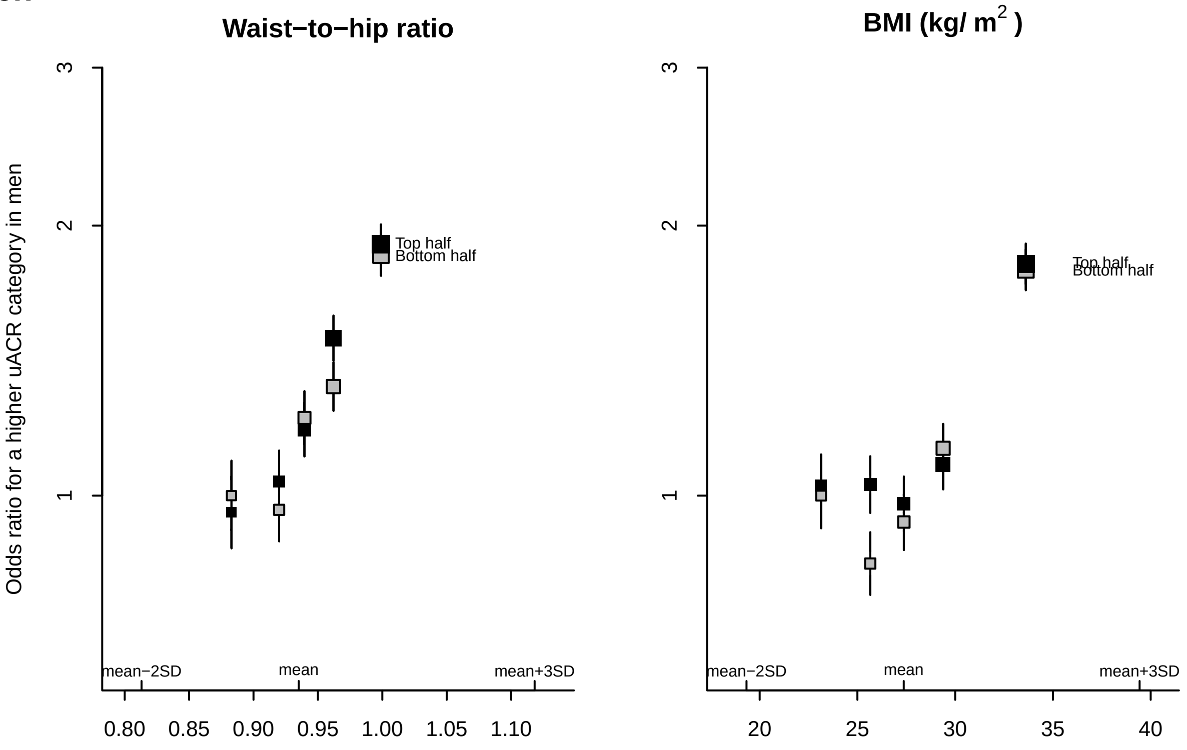

Women

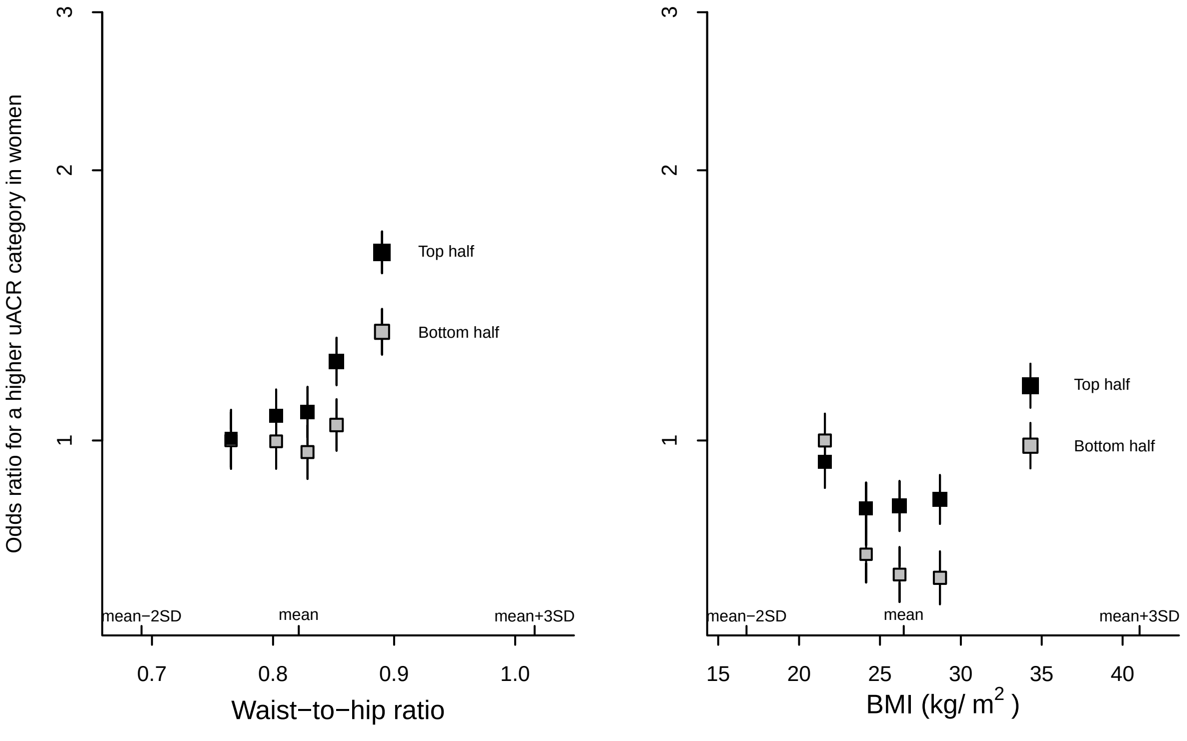

BMI: body mass index.
Model was adjusted for confounders (age, ethnicity, education, region, Townsend deprivation index, smoking, physical activity) and reciprocal adiposity (ie. waist−to−hip ratio adjusted for BMI, BMI adjusted for waist−to−hip ratio) at baseline.
The confounding effect of urinary sodium−to−creatinine ratio was small and was not included in analyses.
ORs (95% CI) per 5 Kg/ m^2 increase in BMI before and after adjustment of urinary sodium−to−creatinine ratio were 1.47 (1.43 − 1.50) and 1.46 (1.43 − 1.50).
ORs (95% CI) per 0.06 increase in waist−to−hip ratio before and after adjustment of urinary sodium−to−creatinine ratio were 1.62 (1.58 − 1.67) and 1.62 (1.57 − 1.67).
